# ADAR1 as a Placental Innate Immune Rheostat Sustaining the Homeostatic Balance of Intrinsic Interferon Response at the Maternal‐Fetal Interface

**DOI:** 10.1002/advs.202505491

**Published:** 2025-08-18

**Authors:** Xiaogang Chen, Xiangchao Xu, Jiahao Chen, Huiru Zhang, Wanshan Zheng, Wenzhe Yu, Xiaoqian Hu, Bin Cao

**Affiliations:** ^1^ Fujian Provincial Key Laboratory of Reproductive Health Research Cancer Research Center Department of Obstetrics and Gynecology Women and Children's Hospital School of Medicine Xiamen University Xiamen Fujian 361102 China; ^2^ State Key Laboratory of Vaccines for Infectious Diseases Xiang An Biomedicine Laboratory School of Public Health Xiamen University Xiamen Fujian 361102 China

**Keywords:** *Adar1*, embryonic lethality, placenta, SINEs, spatial interferon response

## Abstract

The mechanisms that balance a robust intrinsic antiviral defense at the maternal‐fetal interface with fetal development remain elusive. Here, it is delineated that ADAR1, an adenosine‐to‐inosine (A‐to‐I) editor, fine‐tunes intrinsic interferon (IFN)‐mediated integrates stress response (ISR) in the mouse placenta, thereby orchestrating embryonic development and antiviral defense. Placental *Adar1* deletion (*Adar1^PKO^*) trigger spatially resolved overwhelming IFN responses, which impair the differentiation of IFN hyper‐responsive junctional zone (JZ) progenitors and functions of the placental JZ, ultimately causing embryonic death. Mechanistically, the *Adar1^PKO^* placental IFN hyper‐response is positively amplified by the accumulated immunogenic dsRNAs from the 3′UTR of interferon‐stimulated genes (ISG‐3′UTR‐dsRNA). The resulting fetal death is rescued by concurrent deletion of *Mavs*, *Ifnar1*, or *Pkr*, but not *Zbp1* or cell death effectors. Notably, blocking ISR pharmacologically preventes embryonic lethality induced by *Adar1^PKO^* JZ defects. These findings demonstrate that ADAR1 fine‐tunes the unique spatially resolved IFN‐PKR‐ISR signaling in the placenta by restricting ISG‐3′UTR‐dsRNA accumulation, highlighting a potential therapeutic strategy for treating interferonopathy‐associated pregnancy complications.

## Graphical abstract

This study reveals that ADAR1, an RNA‐editing enzyme, fine‐tunes immune responses in the placenta by preventing the accumulation of immunogenic double‐stranded RNAs (dsRNAs) from interferon‐stimulated genes. The loss of ADAR1 in the placenta leads to excessive interferon signaling restricted to the junctional zone, disrupting placental development and causing embryonic death, which is rescued by blocking interferon‐induced integrated stress response (ISR).

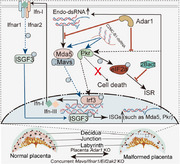

## 1 Introduction

Immune homeostasis at the maternal‐fetal interface is essential for a successful pregnancy and healthy fetal development.^[^ ^1^ ^]^ Throughout pregnancy, the placenta maintains a robust yet controlled intrinsic immune response, restricting vertical pathogen transmission while averting immune‐mediated fetal injury.^[^ ^1^, ^2^, ^3^ ^]^ As critical antiviral defenses, interferons (IFNs, including type I, type II, and type III IFNs) play context‐dependent multifaceted roles during pregnancy.^[^ ^4^ ^]^ IFNs bind to their corresponding receptors and subsequently activate JAK‐STAT signaling pathways, thereby inducing the expression of hundreds of interferon‐stimulated genes (ISGs). In the placenta, IFN secretion and the corresponding ISG induction promote spiral artery remodeling and virus restriction.^[^ ^4^, ^5^ ^]^ On the contrary, elevated IFN levels in interferonopathies including “TORCH” infections (toxoplasmosis, other, rubella, cytomegalovirus, and herpes) and genetic interferonopathies (i.e., Aicardi‐Goutières syndrome (AGS), Down syndrome in trisomy 21, and systemic lupus erythematosus) are linked to pregnancy complications,^[^ ^4^, ^6^, ^7^, ^8^ ^]^ suggesting that IFN activation is a double‐edged sword for a successful pregnancy. Therefore, it is essential to elucidate the key regulatory mechanism of IFN response in the placenta, which sustains the antiviral power of IFNs while preventing maternal or fetal disorders.

In the presence of immunogenic double‐stranded RNA (dsRNA), cells rely on melanoma differentiation‐associated gene 5 (MDA5, encoded by *Ifih1*) to initiate signaling cascades that phosphorylate downstream transcription factors (Irf‐3 and Irf‐7), inducing the production of type I and III IFNs.^[^ ^9^ ^]^ As the first line of immune defense against invading viruses, the placenta empowers a unique mechanism of spontaneous type III IFN production under physiological situations, by recognizing endogenous dsRNAs through constitutively activated MDA5 and dsRNA‐dependent protein kinase R (PKR).^[^ ^3^, ^10^ ^]^ These immunogenic endogenous dsRNAs are specifically derived from placenta‐unique imprinted microRNA clusters with short interspersed nuclear elements (SINEs) in humans or B1 elements (an Alu‐like family of SINEs) in mice. Of note, structurally similar SINE elements with the potential of forming immunogenic dsRNAs are abundantly present within various transcripts.^[^ ^11^ ^]^ However, the mechanisms by which these dsRNAs avert inappropriate recognition by constitutively activated MDA5 and PKR in the placenta remain largely unknown.

A‐to‐I RNA editing, the most common form of post‐transcriptional RNA modification, preferably occurs on SINEs‐derived dsRNAs.^[^ ^12^ ^]^ This process is catalyzed by adenosine deaminase acting on RNA (ADAR) enzymes, including the ubiquitously expressed ADAR1 and the brain‐specific ADAR2, both of which feature conserved dsRNA‐binding domains and a deaminase domain. Additionally, ADAR1 contains Z‐DNA‐binding domains (Z‐α and Z‐β) recognizing Z‐form nucleic acid.^[^ ^13^, ^14^ ^]^ Clinically, mutations in *ADAR1* cause AGS type 6, characterized by overwhelming proinflammatory cytokine production.^[^ ^4^, ^15^ ^]^ As the primary A‐to‐I editor, the cytoplasmic ADAR1p150 isoform specifically hyper‐edits SINE and Alu dsRNAs, preventing the recognition of self‐dsRNAs by MDA5.^[^ ^16^, ^17^, ^18^ ^]^ In murine models, *Adar1* null mice (*Adar1^‐/‐^*) are embryonic lethal at embryonic day (E) 12.5, which are rescued to birth by concurrent deletion of *Ifih1* or the gene of downstream adaptor protein mitochondria‐associated antiviral signaling (*Mavs*).^[^ ^16^, ^17^, ^18^, ^19^ ^]^ A pioneering study has shown that 68% of embryonic lethality between E9.5 and E14.5 was primarily attributed to severe placental malformations.^[^ ^20^ ^]^ These evidences suggest that ADAR1 may maintain homeostatic IFN responses in the placentas and be involved in IFN‐triggered pregnancy disorders.

In this study, we demonstrated that ADAR1 functioned as a regulatory rheostat of placental innate immunity by eliminating the endogenous ISG‐3′UTR‐dsRNA and preventing a unique spatially restricted IFN response in the placenta. Placenta‐specific *Adar1* deficiency triggered the activation of the dsRNA‐MAVS‐IFN‐PKR‐integrated stress response (ISR) signaling axis specifically in placental junctional zone (JZ) trophoblasts and subsequently caused fetal lethality. Mechanistically, deletion of *Adar1* cyclically overloaded the ISG‐derived 3′ UTR‐dsRNAs onto the constitutively active type III IFN driving sensors MDA5 and PKR in the placenta, thereby shifting well‐controlled antiviral machinery to a toxic IFN response. Notably, inhibition of the ISR with the small molecule 2BAct alleviated JZ defects in *Adar1^PKO^* placentas and corresponding embryonic lethality.

## 2 Results

### 2.1 Placenta‐Specific *Adar1* Deletion Triggers Fetal Demise at E14.5 Caused by Striking JZ Defects

**Figure 1:**
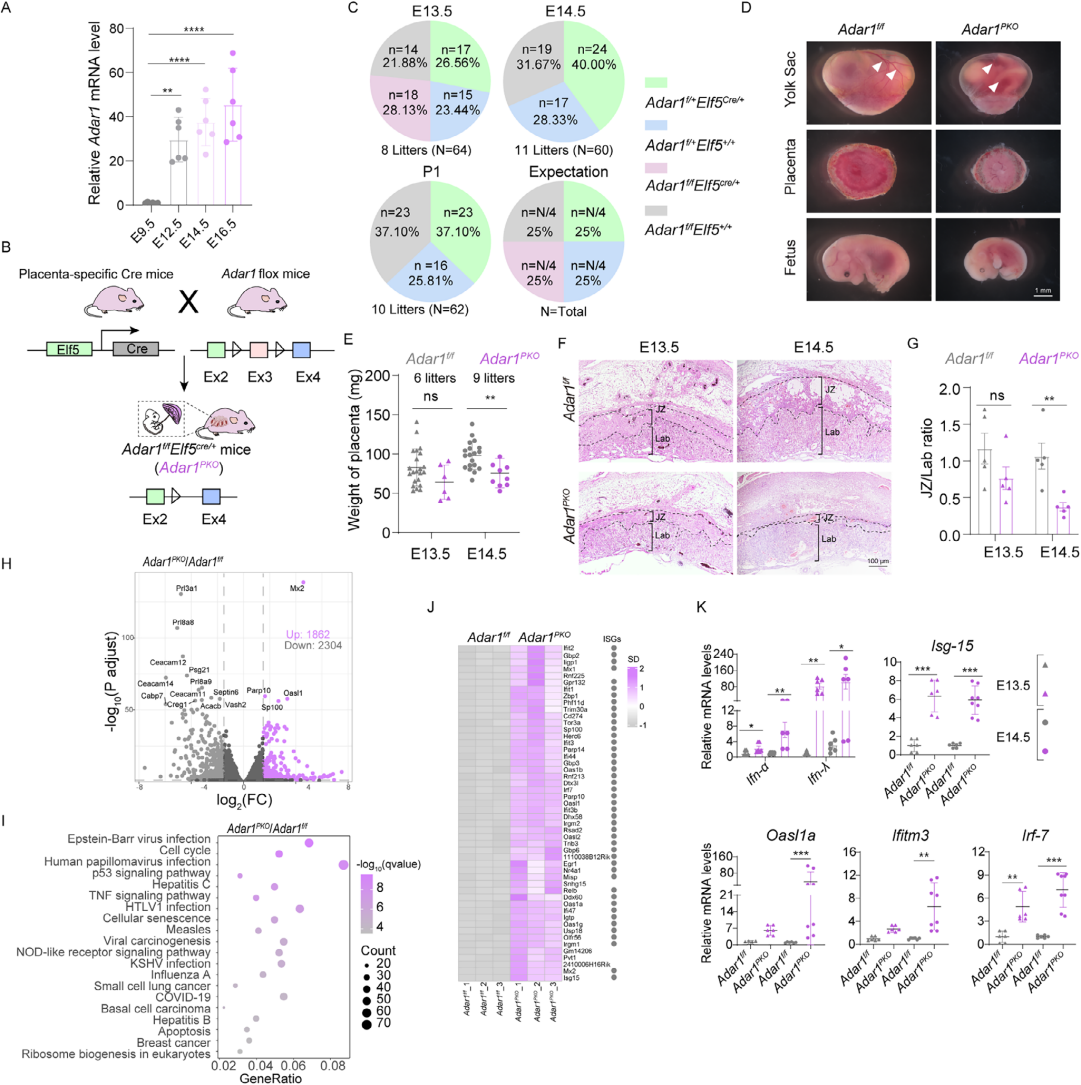
Specific *Adar1* deletion in the placenta triggers sudden fetal demise at E14.5 featured by striking JZ defects. A) Relative expression of *Adar1* in placentas (n = 6, 6 litters). B) Schematic of the mating strategy for placenta‐specific *Adar1* knockout mice (*Adar1^PKO^*). C) Genetic analysis of embryos from *Adar1^f/f^* dams mated to *Adar1^f/+^Elf5^Cre/+^* sires at E13.5 and E14.5. D) Representative images of the yolk sac, placenta, and fetus from E14.5 *Adar1^f/f^* and *Adar1^PKO^* embryos. Arrowhead, yolk sac vasculature. E) Weights of *Adar1^f/f^* and *Adar1^PKO^* placentas. Dots represent individual mice at E13.5 and E14.5. F) Representative H&E staining showing the histology of *Adar1^f/f^* and *Adar1^PKO^* placentas at E13.5 and E14.5. G) Quantification of the JZ/Lab ratio in the indicated placentas (n = 5, 5 litters). H) Volcano plots depict significant DEGs detected by RNA‐seq in *Adar1^PKO^* versus *Adar1^f/f^* placentas at E14.5. I) GO analysis of the upregulated DEGs in *Adar1^PKO^* placentas at E14.5. J) Heatmap represents relative expression of the top 50 upregulated genes in *Adar1^PKO^* placentas at E14.5. The ISGs annotated in the Interferome dataset [http://interferome.its.monash.edu.au/interferome/] are checked by grey circles. K) Relative expression of IFNs and ISGs in placentas (n = 8, 5 litters). In panels A, E, G, and K, Data are presented as mean ± SEM. *p < 0.05, **p < 0.01, ***p < 0.001; ns, non‐significant; unpaired t test was used.

A recent study demonstrated that the human and mouse placenta constitutively produced type III IFN,^[^ ^10^ ^]^ which was triggered by placental SINE‐derived dsRNAs. Here, we showed that both *Ifnb* and *Ifnl* progressively increased in mouse placentas from E9.5 to E16.5 (Figure S1A,B, Supporting Information). Intriguingly, the concomitant increased expression of *Adar1*, the prominent SINE‐dsRNA editor, was observed as pregnancy advanced (**Figure** **1**A), suggesting that ADAR1 may respond to dynamically changed intrinsic dsRNAs, maintaining a homeostatic intrinsic IFN response.

To assess the intrinsic effect of ADAR1 in the placenta, we intercrossed *Adar1^f/f^* mice with *Elf5‐Cre* mice to generate trophoblast‐specific *Adar1* KO mice (herein referred to as *Adar1^PKO^*) (Figure 1B). *Elf5‐Cre* mediated recombination resulted in the deletion of exon 3 of *Adar1* (encoding the dsRNA binding domain necessary for A‐I editing) specifically in the placenta, which was verified by single‐cell resolution in situ hybridization on tissues (SCRINSHOT) staining and Western blot (Figure S2A,C, Supporting Information). Consistently, Sanger sequencing of complementary DNA reverse‐transcribed from *Rpa1*, *Mad2l1*, and *Rbbp4* mRNA, the established ADAR1 substrates, confirmed defective ADAR1 activity in *Adar1^PKO^* placentas (Figure S2B, Supporting Information). Intriguingly, *Adar1^PKO^* fetuses did not survive to birth and died at E14.5 (Figure 1C). *Adar1^PKO^* fetuses exhibited growth retardation and pale yolk sacs devoid of fetal blood, compared to the *Adar1^f/f^* littermates (Figure 1D). Furthermore, the weights of *Adar1^PKO^* placentas and embryos were significantly reduced (Figure 1E; Figure S2D, Supporting Information), manifesting signs of growth delay in *Adar1^PKO^* mice. Consistently, H&E staining revealed a reduced thickness of the JZ layer in *Adar1^PKO^* placentas (Figure 1F,G). This evidence suggests that *Adar1* deletion in the placenta causes a defective placental JZ layer and subsequently embryonic lethality at E14.5.

To investigate the transcriptome signatures underlying embryonic lethality and placental development disorders, we then performed RNA sequencing (RNA‐seq) on *Adar1^PKO^* placentas at both E13.5 and E14.5. We identified 1572 differentially expressed genes (DEGs) (783 upregulated and 789 downregulated genes) and 4166 DEGs (1862 upregulated and 2304 downregulated genes) at E13.5 (Figure S2E, Supporting Information) and E14.5 (Figure 1H), respectively. Gene ontology (GO) analysis highlighted consistent antiviral pathways induced by the alteration of *Adar1*, including pathways related to Epstein‐Barr virus infection, Human papillomavirus infection, Hepatitis C, and others (Figure 1I; Figure S2F, Supporting Information). Likely, most of the upregulated DEGs, especially at E14.5, were identified as ISGs, such as *Irf7, Isg‐15, Oasl1a*, and *Ifitm3* (Figure 1J; Figure S2G,H, Supporting Information). We further examined the expression profiles of inflammatory genes, IFNs, and the aforementioned classical ISGs in placentas at E13.5 and E14.5. RT‐qPCR revealed a robust upregulation of *Ifna1*, *Ifnl2*, *Il‐6*, and selected ISGs in the *Adar1^PKO^* placenta, while *Il‐1β* and *Tnf‐α* levels remained unchanged (Figure 1K; Figure S2I, Supporting Information). Taken together, these data indicate that the *Adar1* deletion in the placenta spontaneously initiates sterile inflammation, causing JZ defects and subsequently embryonic lethality at E14.5.

### 2.2 Spatially Resolved IFN Responses Induced by Placental *Adar1* Deletion Impair JZ Morphogenesis and Functions

**Figure 2:**
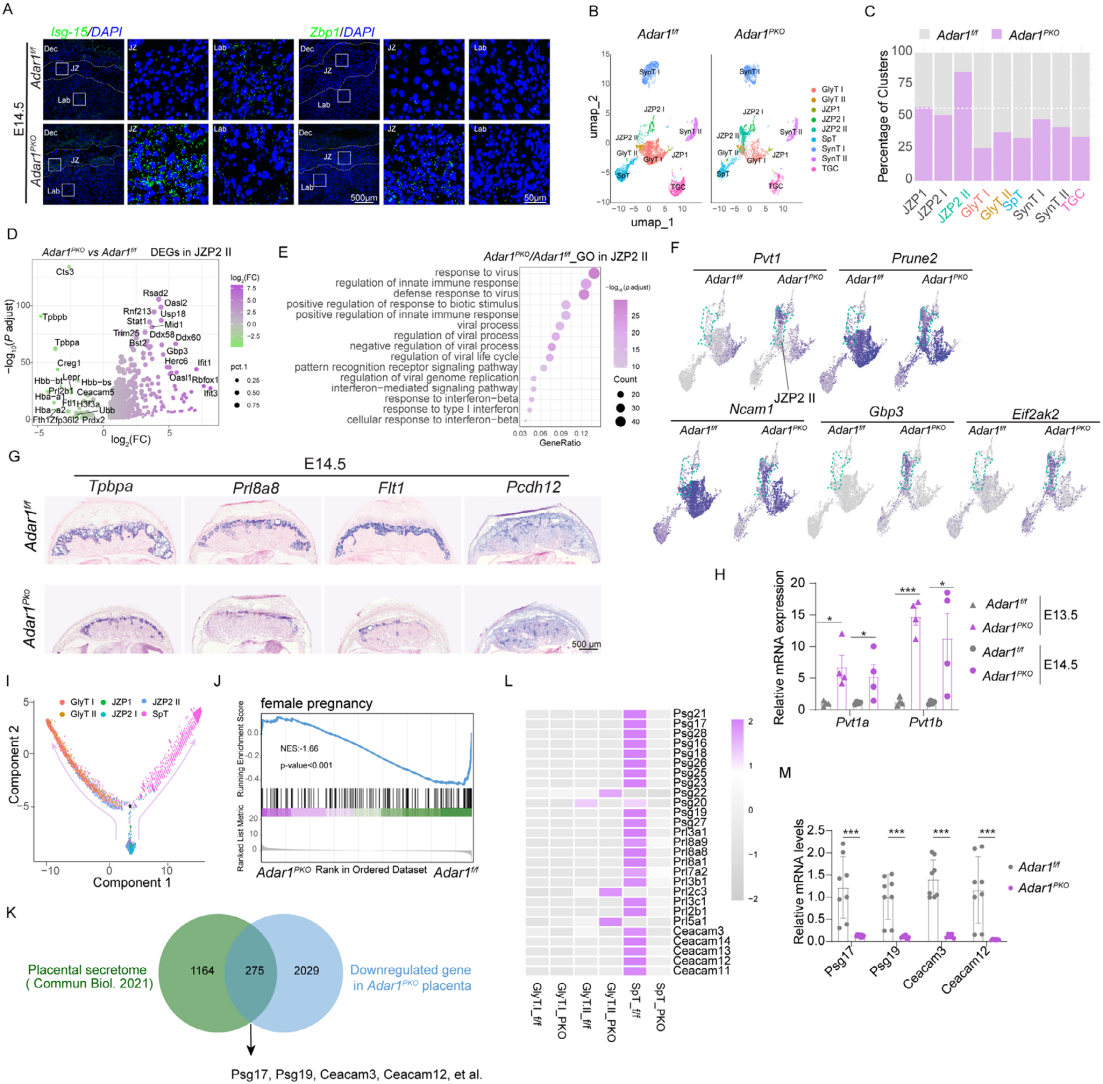
JZP‐specific IFN responses induced in *the Adar1* deletion placenta impaired JZ morphogenesis and functions. A) SCRINSHOT for *Isg15* and *Zbp1* mRNA in *Adar1^f/f^* and *Adar1^PKO^* placentas at E14.5. B) UMAP visualization of the mouse trophoblastic population in *Adar1^f/f^* and *Adar1^PKO^* placentas at E14.0. C) Quantification of cluster composition of the mouse trophoblast cells by normalizing for the total number of cells per sample. D) Volcano plot of DEGs in JZP2 II from the *Adar1^PKO^* placentas relative to the *Adar1^f/f^* counterparts. E) GO analysis of significantly upregulated genes (*Adar1^PKO^* vs *Adar1^f/f^*) in JZP2 II. F) Feature plot showing the expression of junctional zone progenitor markers (*Pvt1*, *Prune2*, and *Ncam1*) and highly expressed ISGs (*Gbp3* and *Eif2ak2*) in JZP2 II of *Adar1^f/f^* and *Adar1^PKO^* placentas. Green circle, JZP2 II. G) Representative ISH analysis of four JZ markers (*Tpbpa*, *Prl8a8*, *Flt1*, and *Pcdh12*). H) RT–qPCR analysis of relative mRNA expression of *Pvt1a* and *Pvt1b* in *Adar1^f/f^* and *Adar1^PKO^* placentas at E13.5 and E14.5 (n = 4, 4 litters). I) Pseudotime ordering reveals IJZP with two branches to the GlyT and the SpT from *Adar1^f/f^* and *Adar1^PKO^* placentas. J) GSEA of downregulated genes in *Adar1^PKO^* placentas identified by RNA‐seq highlighting the defects of the female pregnancy pathway. K) Venn diagram showing the overlap of downregulated genes in *Adar1^PKO^* placentas with previously identified placental secretome genes. L) Heatmap illustrating the relative expression of the significantly downregulated genes involved in placental secretome in JZ trophoblasts (GlyT and SpT) at E14.0. M) RT–qPCR analysis of relative mRNA expression of pregnancy‐specific secreted glycoproteins in *Adar1^f/f^* and *Adar1^PKO^* placentas (n = 8, 5 litters). In panels H and M, Data are presented as mean ± SEM. *p < 0.05, ***p < 0.001; unpaired t test was used.

Given that the pathological changes of *Adar1^PKO^* placentas were confined to the JZ layer, we interrogated spatially resolved IFN responses in the placenta induced by *Adar1* deletion. SCRINSHOT staining and in situ hybridization (ISH) revealed that representative ISGs (*Isg‐15*, *Zbp1* and *Ifitm3*) were mainly expressed in the JZ of *Adar1^PKO^* placentas (**Figures** **2**A and S3A, Supporting Information), supporting a spatially restricted IFN response. Motivated by this result, we performed single‐nuclei RNA sequencing (snRNA‐seq) on *Adar1^PKO^* placentas at E14.0 to further dissect the heterogeneous cellular responses to Adar1 deficiency across different trophoblast subtypes. By manually combining clusters using trophoblast markers via UMAP visualization, we identified nine subgroups: junctional zone precursor 1 (JZP1), JZP2 I, JZP2 II, glycogen trophoblast I (GlyT I), GlyT II, spongiotrophoblast (SpT), SynT I, SynT II, and TGC (Figure 2B). Notably, five clusters exhibited significant alterations in cell numbers due to *Adar1* deletion, including four decreased clusters (GlyT I, GlyT II, SpT, and TGC) and the increased JZP2 II (Figure 2C). Among the nine populations, genes associated with IFN signaling were predominantly expressed in JZP2 II, especially *Ifih1* (encoding MDA5, a dsRNA sensor), *Irf‐7* (a transcription factor of IFNs), *Stat2*, *Stat1*, and *Irf‐9* (transcription factors driving ISGs expression), suggesting elevated IFN response predominantly in the JZP2 II (Figure 2D; Figure S3B, Supporting Information). GO analysis further confirmed robust IFN response signatures in the JZP2 II (Figure 2E). Additionally, ISGs, such as *Gbp3* and *Eif2ak2*, were exclusively expressed in JZP2 II (Figure 2D,F). Based on these results, we defined JZP2 II as an IFN‐responsive junctional zone progenitor (IJZP) in the *Adar1^PKO^* placenta. These findings imply that excessive IFN response in IJZP may be the initial cause of JZ disruption in the *Adar1^PKO^* placenta.

Next, we analyzed the expression of terminally differentiated trophoblast markers, the spatial expression of labyrinth trophoblast markers, including *Ctsq* (an sTGC marker), Mct1 (SynT I), and Mct4 (SynT II), showed no difference in *Adar1^PKO^* placentas (Figure S3C,D, Supporting Information). Of note, ISH illustrated that the expression of mature JZ trophoblast markers, including *Tpbpa*, *Prl8a8*, *Flt1*, and *Pcdh12*, were barely observed in JZ upon selective *Adar1* deletion in placentas (Figure 2G; Figure S3D, Supporting Information). Conversely, the expression of *Pvt1*, an IJZP marker, was elevated in the *Adar1^PKO^* placenta, in line with the increased cell number of IJZP observed in snRNA‐seq data (Figure 2C,F,H), implying that a strong IFN response may impede the IJZP‐to‐mature JZ differentiation. Indeed, RNA velocity analysis revealed that IJZP branched into the GlyT and SpT lineage through two distinct trajectories (Figure 2I), suggesting that the malformed JZ layer in the *Adar1^PKO^* placenta may be attributed to improper IJZP differentiation into mature JZ trophoblast lineages.

Considering the markedly compromised JZ trophoblast differentiation, we speculated that specific JZ functions, such as glycogen storage and the secretion of pregnancy‐associated proteins,^[^ ^21^ ^]^ were impaired in the *Adar1^PKO^* placenta. As expected, Periodic acid‐Schiff (PAS) staining corroborated reduced glycogen storage in the *Adar1^PKO^* placenta (Figure S3E, Supporting Information). Gene Set Enrichment Analysis (GSEA) and GO terms of bulk RNA‐seq data revealed that the downregulated DEGs in *Adar1^PKO^* placentas were enriched in pathways associated with female pregnancy and N‐Glycan biosynthesis (Figure 2J; Figure S3F,G, Supporting Information). Consistently, 275 genes were previously identified as coding genes for placenta‐secreted proteins (Figure 2K). Emerging evidence has shown that multiple pregnancy disorders^[^ ^22^ ^]^ and SARS‐CoV‐2 infection in the human placenta are characterized by declined pregnancy‐specific glycoproteins (PSG) levels (Figure S3H,I, Supporting Information). RT‐qPCR and snRNA‐seq data confirmed that the expression of PSGs (*Psg17, Psg19, Ceacam3*, and *Ceacam12*) was significantly reduced in JZ trophoblasts of the *Adar1^PKO^* placenta (Figure 2L,M), supporting the notion that JZ functions were compromised in these placentas. In summary, our results suggest that the differentiation and functions of JZ trophoblasts are specifically disrupted due to spatially enriched IFN hyper‐response caused by *Adar1* deficiency.

### 2.3 MAVS, but not ZBP1, is Indispensable for IFN Induction in the *Adar1^PKO^* Placenta

**Figure 3:**
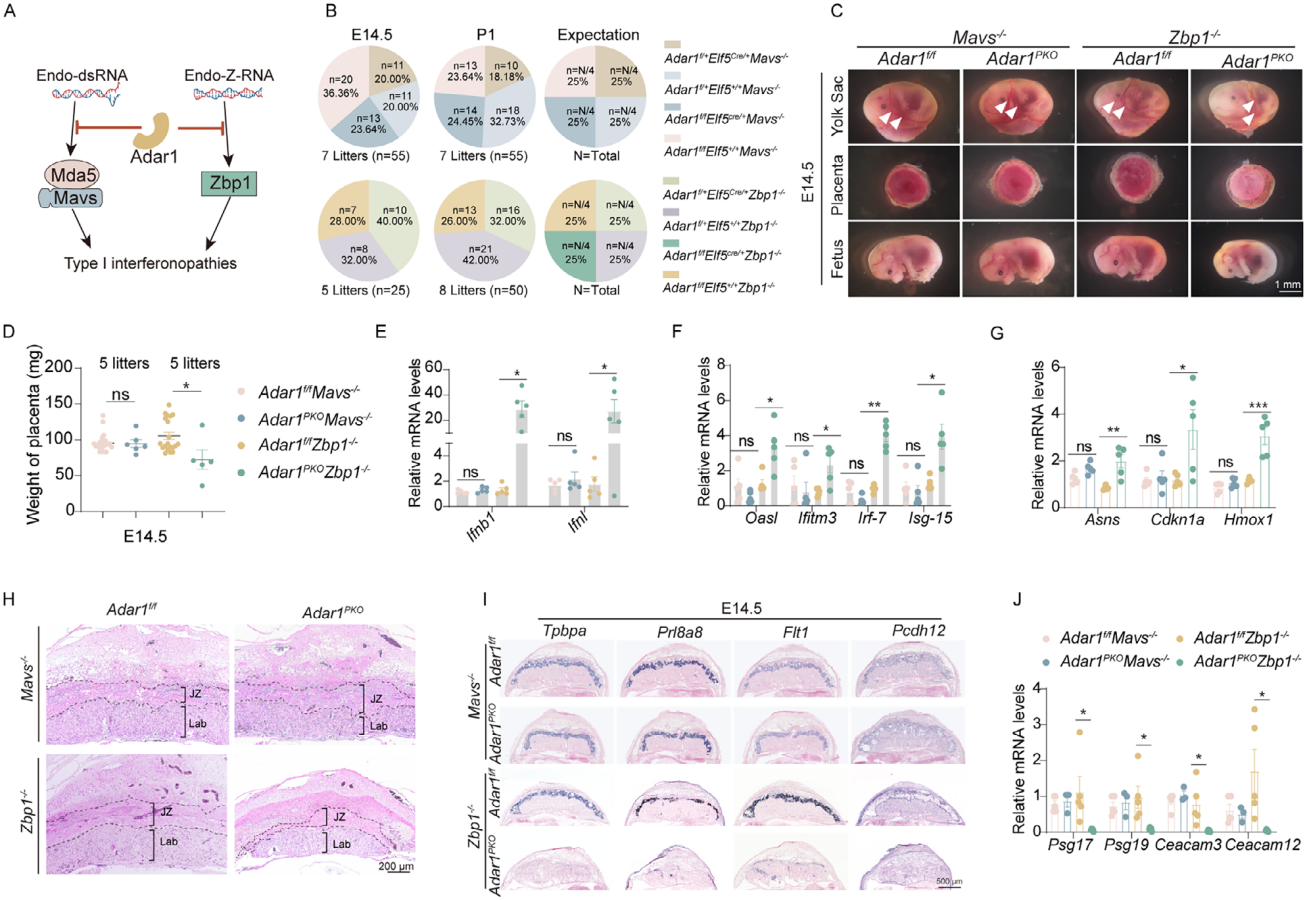
IFN induction in the *Adar1^PKO^* placenta is dependent on MAVS rather than ZBP1. A) Schematic illustrates the role of ADAR1, MAVS, and ZBP1 in modulating IFN responses induced by endogenous dsRNAs and Z‐RNAs. B) Genotypic distribution of progenies at E14.5 and P1 from *Adar1^f/f^Mavs^‐/‐^* or *Adar1^f/f^Zbp1^‐/‐^* dams mated to *Adar1^f/+^Elf5^Cre/+^Mavs^‐/‐^ or Adar1^f/+^Elf5^Cre/+^Zbp1^‐/‐^* sires, respectively. C) Morphology of the yolk sac, placenta, and fetus at E14.5. Arrowheads, yolk sac vasculature. D) Placenta weights of embryos from indicated genotypes at E14.5. Dots represent individual placentas. E‐G) RT–qPCR analysis of relative mRNA expression levels of IFNs E), ISGs F), and selected ISR markers G) in E14.5 placentas (n = 6, 5 litters). H) H&E staining of placentas with indicated genotypes at E14.5. I) ISH analysis of JZ markers (*Tpbpa*, *Prl8a8*, *Flt1*, and *Pcdh12*) in E14.5 placentas. J) RT–qPCR showing the expression of pregnancy‐specific secreted glycoproteins in E14.5 placentas (n = 6, 5 litters). In panels D, E, F, G, and J, Data are presented as mean ± SEM. *p < 0.05, **p < 0.01, ***p < 0.001; ns, non‐significant; unpaired t test was used.

We next investigated the functional relevance of the critical RNA sensing pathway to spontaneous IFN responses and JZ defects in the *Adar1^PKO^* placenta. Since *Adar1* deficiency can result in the accumulation of dsRNAs or Z‐RNAs, which are respectively recognized by MDA5‐MAVS or ZBP1 (**Figure** **3**A),^[^ ^23^ ^]^ we assessed whether MAVS or ZBP1 drives the IFN overproduction in the *Adar1^PKO^* placenta. To test this, we mated *Adar1^f/f^Mavs^‐/‐^* or *Adar1^f/f^Zbp1^‐/‐^* mice with *Adar1^f/+^Elf5^Cre/+^Mavs^‐/‐^* or *Adar1^f/+^Elf5^Cre/+^Zbp1^‐/‐^* mice to generate *Adar1^PKO^Mavs^‐/‐^* or *Adar1^PKO^Zbp1^‐/‐^* mice (Figure S4A, Supporting Information). Similar to *Adar1^PKO^* mice, the *Adar1^PKO^Zbp1^‐/‐^* fetus died at E14.5 and exhibited severe phenotypic defects (Figure 3B,C), indicating that the ZBP1 pathway is dispensable for the embryonic lethality associated with placenta‐specific *Adar1* deficiency. On the contrary, *Adar1^PKO^Mavs^‐/‐^* offspring were observed at statistically normal frequencies according to Mendelian ratios (Figure 3B). In line with this, the *Mavs* deletion completely restored placental and embryonic weights, normalized fetus size, and prevented the IFN production and ISG upregulation in the placenta (Figure 3C–F; Figure S4B–D, Supporting Information). In addition to the IFN pathway, *Adar1* loss has been shown to promote MDA5‐PERK‐α subunit of eukaryotic initiation factor 2 (eIF2α) pathway‐dependent stress response.^[^ ^24^ ^]^ Therefore, we measured the expression of three canonical ISR genes (*Asns, Cdkn1a*, and *Hmox1*) in placentas. RT‐qPCR showed that *Adar1* loss in placentas led to robust induction of ISR transcripts, which is dependent on MAVS but not ZBP1 (Figure 3G). Consistent with the changes in ISG and ISR signatures, histological defects were evident in *Adar1^PKO^Zbp1^‐/‐^* placentas, but were prominently rescued in *Adar1^PKO^Mavs^‐/‐^* placentas (Figure 3H–I; Figure S4E, Supporting Information). Moreover, the mRNA expression of four representative PSG genes in the *Adar1^PKO^Mavs^‐/‐^* placenta was restored to control levels, whereas their expression was still maintained low in the *Adar1^PKO^Zbp1^‐/‐^* placenta (Figure 3J), indicating that the defective secretory function associated with IFN activation is dependent on MAVS. In summary, these data demonstrate that MAVS, but not ZBP1, is necessary for mediating the endogenous excessive IFN response in the placenta, which causes embryonic lethality in *Adar1^PKO^* mice.

### 2.4 Loss of *Adar1* Results in the Accumulation of ISGs‐Derived Endogenous dsRNAs by Positive Feedback

**Figure 4:**
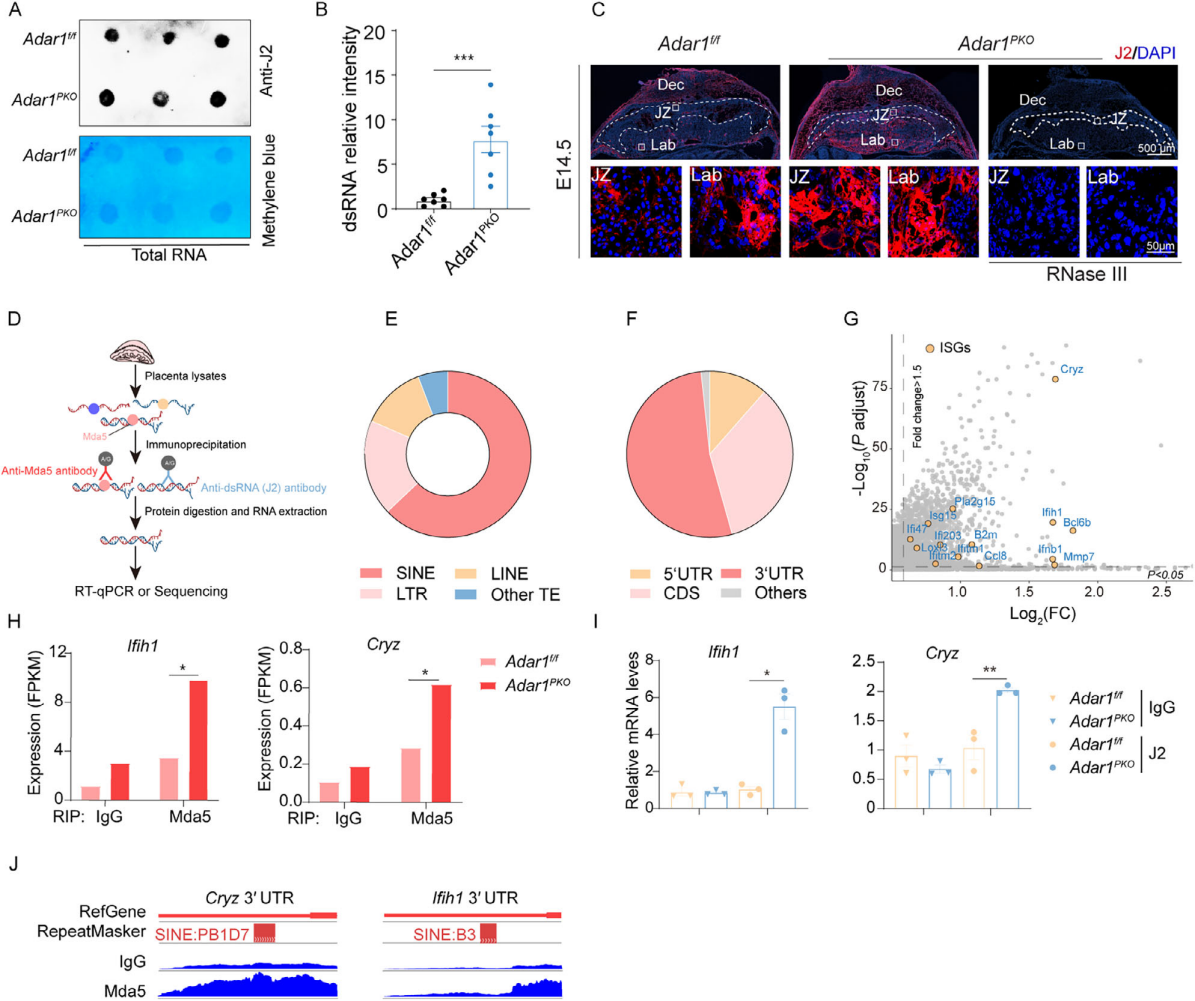
Loss of *Adar1* results in the accumulation of ISGs mRNA‐derived endogenous dsRNAs by positive feedback. A,B) Dot blot A) and the corresponding quantification B) of dsRNA levels in *Adar1^f/f^* and *Adar1^PKO^* placentas (n = 6, 6 litters). Methylene blue staining was used to confirm equal RNA loading. C) Representative immunofluorescence staining for dsRNA using the J2 antibody in E14.5 placentas. RNase III treatment (a specific RNase digesting dsRNA) was used to verify the identification of dsRNA. D) Schematic illustrates MDA5‐RIP‐Seq or J2‐RIP RT‐qPCR of placentas. E) Proportion of retrotransposon elements (SINE, LINE, LTR, and other TEs) in MDA5‐RIP enriched RNAs in *Adar1^PKO^* placentas. LTR: Long Terminal Repeat. LINE: Long Interspersed Elements. SINE: Short Interspersed Elements F) Genomic distribution of MDA5‐RIP enriched RNAs in the 5′UTR, coding sequence (CDS), 3′UTR, and other regions of genes from *Adar1^PKO^* versus *Adar1^f/f^* placentas. G) Scatter plots show ISG mRNAs enriched by MDA5‐RIP‐seq in E14.0 *Adar1^PKO^* placentas. H,I) Expression of ISGs (*Ifih1* and *Cryz*) identified by MDA5‐RIP‐seq (H) and J2 RIP‐RT‐qPCR I) in *Adar1^PKO^* versus *Adar1^f/f^* placentas. (H, n = 2 biological replicates per group; I, 3 litters, n = 3). J) Representative MDA5 binding loci in the indicated ISG mRNA transcripts (*Cryz* and *Ifih1*) in *Adar1^PKO^* placentas. The red square represents the location of SINEs. In panels B, H, and I, Data are presented as mean ± SEM. *p < 0.05, **p < 0.01, ***p < 0.001; unpaired *t* test was used.

As ADAR1 destabilizes complementary inverted Alus duplexes,^[^ ^25^, ^26^ ^]^ we hypothesized that *Adar1* loss may result in the aberrant formation of endogenous dsRNA bound by MDA5. To test this, we performed a dot blot assay using the J2 antibody, which specifically recognizes dsRNAs, and found that *Adar1* deficiency in the placenta induced a remarkable dsRNA accumulation (**Figure** **4**A,B). Consistent with the strong IFN responses and placental dysfunctions in the JZ of *Adar1^PKO^* placentas, the accumulated endogenous dsRNA signal was primarily evident in the JZ (Figure 4C; Figure S5A, Supporting Information). Worth to note, control placentas also exhibited J2 staining signal, but with much lower intensity. In summary, these data suggest that the spatially enriched dsRNAs in the *Adar1^PKO^* placenta JZ act as the trigger for aberrant IFN responses.

To further characterize these dsRNAs excessively enriched in the *Adar1^PKO^* placenta, we performed MDA5 RNA immunoprecipitation (MDA5‐RIP) followed by deep sequencing (Figure 4D). Intriguingly, more than 50% of the MDA5‐enriched RNAs contained SINE repetitive elements that readily form dsRNAs (Figure 4E). Moreover, MDA5‐RIP peaks were enriched in 3′ untranslated regions (3′UTRs) (Figure 4F), implying that 3′UTRs of mRNAs may potentially generate immunostimulatory dsRNAs. Similar to the Z22 (an antibody against Z‐form nucleic acid) RIP results of immortalized *Adar1^‐/‐^Zbp1^‐/‐^* mouse embryo fibroblasts treated with IFN‐β,^[^ ^27^ ^]^ MDA5‐enriched RNAs were characterized as mRNA transcripts of ISGs (Figure 4G; Figure S5B, Supporting Information). Subsequently, we selected two of these ISG mRNAs with 3′UTR SINEs, *Cryz* and *Ifih1*, for further investigation. Both mRNAs were more efficiently pulled down in the *Adar1^PKO^* placenta using MDA5 and J2 antibodies, compared to the IgG controls (Figure 4H,I). Secondary structure prediction of *Cryz* and *Ifih1* mRNAs further suggested the presence of 3′UTR SINE repeats with a high potential for dsRNA formation (Figure 4J; Figure S5C,D, Supporting Information). These findings indicate that ISG 3′UTRs are a prominent source of endogenous immunogenic dsRNA recognized by MDA5 in response to *Adar1* deficiency, which could persistently amplify IFN signaling.

Notably, although *Elf5‐Cre* initiates gene deletion from the blastocyst stage (E4.5),^[^ ^28^ ^]^ the overactivated IFN signaling is not detected until E13.5 in *Adar1^PKO^* placentas (Figure S3A, Supporting Information). Based on the above observation that ISG mRNAs can form immunogenic dsRNAs, we reasoned that *Adar1* deficiency fine‐tunes a pregnancy stage‐dependent immune response to self‐dsRNAs, transitioning from tolerance at early pregnancy to triggering placental damages at E14.5, which might be attributed to the accumulative level of ISGs‐derived dsRNAs. Of note, the progressively increased expression of ISGs (*Isg‐15*, *Oasl1a*, *Irf7*, and *Ifitm3*) as pregnancy advanced peaked at E14.5, coinciding with the time of *Adar1^PKO^* fetal death (Figure S6A,B, Supporting Information). Taken together, these results suggest that ISGs containing 3′UTR dsRNAs, which are constitutively expressed in the placenta and induced by *Adar1* deficiency, synergistically amplify the initial IFN signaling by positive feedback, ultimately causing JZ defects and sudden embryonic lethality.

### 2.5 *Ifnar1* Deletion Fully Rescues the Embryonic Lethality of *Adar1^PKO^* Mice but does not Alleviate Fetal Growth Restriction

**Figure 5:**
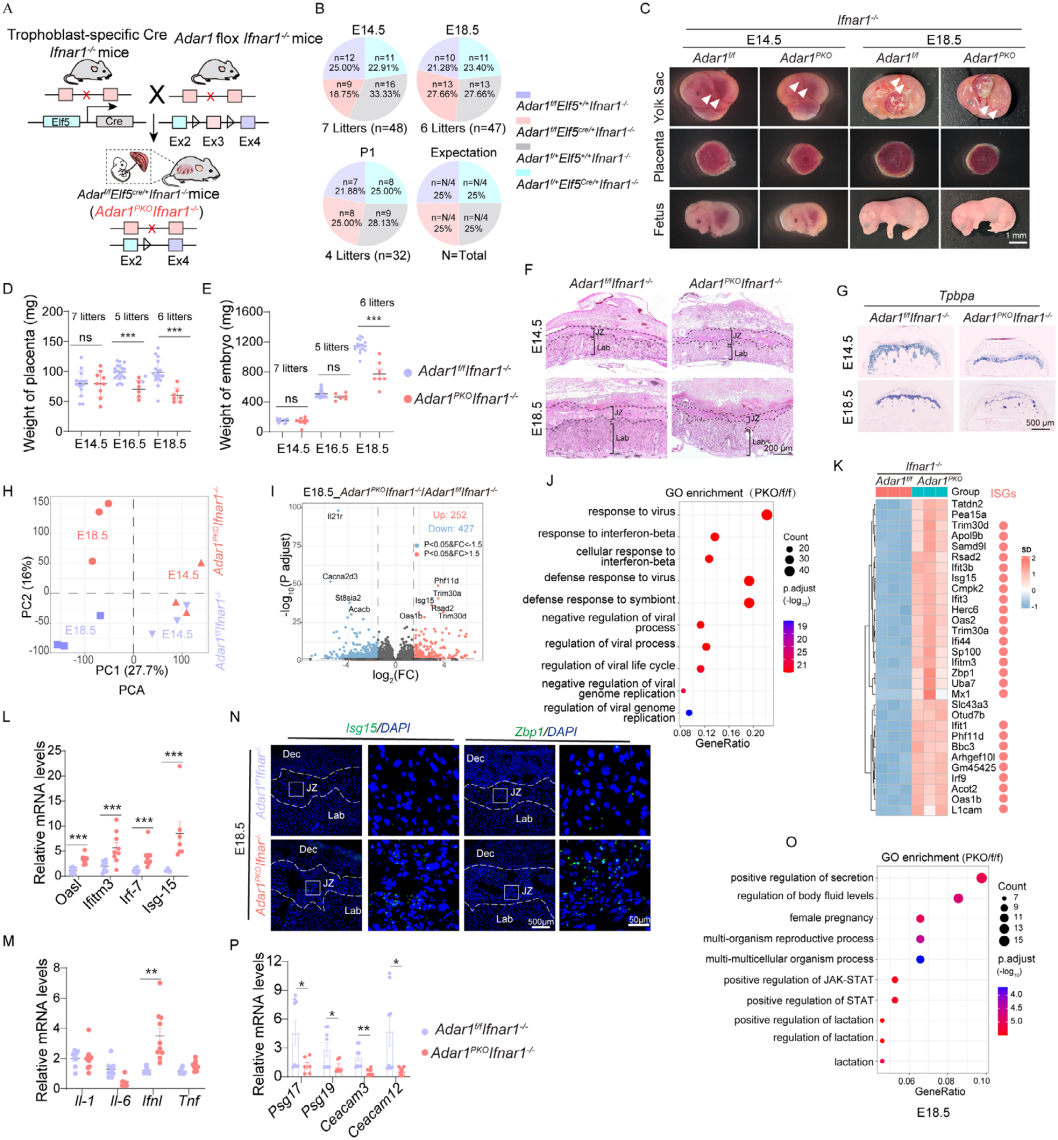
Role of type I IFN in mediating the effects of *Adar1* ablation on placental and fetal development. A) Mating strategy of generating trophoblast‐specific *Adar1* deletion on the *Ifnar1^‐/‐^* background. B) Genotypic distribution of embryos at E14.5, E18.5, and P1 from *Adar1^f/f^Ifnar1^‐/‐^* dams mated to *Adar1^f/+^Elf5^Cre/+^Ifnar1^‐/‐^* sires. C) Stereomicroscopic images of the yolk sac, placenta, and fetus of the indicated genotypes at E14.5 and E18.5. Arrowheads, yolk sac vasculature. D,E) Placental D) and Embryonic E) weight of *Adar1^f/f^Ifnar1^‐/‐^* and *Adar1^PKO^Ifnar1^‐/‐^* embryos at E14.5, E16.5, and E18.5, respectively. Dots represent individual mice. F) H&E staining of *Adar1^f/f^Ifnar1^‐/‐^* and *Adar1^PKO^Ifnar1^‐/‐^* placenta at E14.5 and E18.5. G) ISH of JZ markers in *Adar1^f/f^Ifnar1^‐/‐^* and *Adar1^PKO^Ifnar1^‐/‐^* placentas at E14.5 and E18.5. H) PCA of differentially expressed genes from RNA‐seq data of *Adar1^f/f^Ifnar1^‐/‐^* and *Adar1^PKO^Ifnar1^‐/‐^* placentas at E14.5 and E18.5 (n = 3, 3 litters). I) Volcano plots show differentially expressed genes detected by RNA‐seq of whole *Adar1^f/f^Ifnar1^‐/‐^* and *Adar1^PKO^Ifnar1^‐/‐^* placentas at E18.5. J) GO analysis of significantly upregulated genes in *Adar1^PKO^Ifnar1^‐/‐^* placentas at E18.5. K) Heatmap represents relative expression of the top 30 upregulated genes in *Adar1^PKO^Ifnar1^‐/‐^* placentas at E18.5. Genes featured as the Interferome dataset‐verified ISGs are indicated by the orange circle. L,M) RT–qPCR analysis of relative mRNA expression of ISGs L) and inflammatory genes M) of *Adar1^f/f^Ifnar1^‐/‐^* and *Adar1^PKO^Ifnar1^‐/‐^* placentas at E18.5 (n = 10, 6 litters). N) SCRINSHOT analysis shows *Isg15* and *Zbp1* mRNA in *Adar1^f/f^Ifnar1^‐/‐^* and *Adar1^PKO^Ifnar1^‐/‐^* placentas at E18.5. O) GO analysis of significantly downregulated genes in *Adar1^PKO^Ifnar1^‐/‐^* placentas. P) Relative mRNA expression of pregnancy‐specific secreted glycoproteins in *Adar1^f/f^Ifnar1^‐/‐^* and *Adar1^PKO^Ifnar1^‐/‐^* placentas at E18.5 (n = 6, 5 litters). In panels D, E, L, M, and P, Data are presented as mean ± SEM. *p < 0.05, **p < 0.01, ***p < 0.001; ns, non‐significant; unpaired t test was used.

In light of the excessive ISG induction following placental *Adar1* depletion, we next explored whether deletion of the type I IFN receptor (*Ifnar1* ^‐/‐^) could rescue the phenotype of *Adar1^PKO^* mice. Thus, we crossed *Adar1^PKO^* mice to the genetic background of *Ifnar1^‐/‐^* mice (**Figure** **5**A). Genotype examination of the fetuses (E14.5 and E18.5) and live newborns at birth demonstrated that *Ifnar1* knockout completely prevented the embryonic death of *Adar1^PKO^* mice (Figure 5B). However, detailed phenotypic analyses revealed that the size and weight of *Adar1^PKO^Ifnar1^‐/‐^* fetus, as well as the corresponding placental weight, significantly reduced as the pregnancy progressed beyond E14.5 (Figure 5C–E; Figure S7A,B, Supporting Information), indicating signs of fetal growth restriction (FGR) in the *Adar1^PKO^Ifnar1^‐/‐^* mice. Notably, although the thickness of JZ was rescued at E14.5, histological analysis of the *Adar1^PKO^Ifnar1^‐/‐^* placentas at E18.5 revealed a smaller JZ area, accompanied by remarkable GlyT and SpT loss (Figure 5F,G).

To decipher the molecular basis of these fetal and placental development disorders, we performed bulk RNA sequencing (RNA‐seq) comparing the transcriptomes of *Adar1^f/f^Ifnar1^‐/‐^* versus *Adar1^PKO^Ifnar1^‐/‐^* placentas at both E14.5 and E18.5. Principal components analysis (PCA) showed that while the placental transcriptomes of *Adar1^PKO^Ifnar1^‐/‐^* were not significantly altered at E14.5, they diverged notably from *Adar1^f/f^Ifnar1^‐/‐^* at E18.5 (Figure 5H). Consistently, only 78 upregulated and 94 downregulated genes were identified in *Adar1^PKO^Ifnar1^‐/‐^* placenta at E14.5 (Figure S7C, Supporting Information), compared to 252 upregulated and 427 downregulated DEGs at E18.5 (Figure 5I). GO analysis of these upregulated DEGs revealed significant enrichment of innate immunity pathways, such as responses to the virus, defense response to symbiont, and response to IFN, at both E14.5 and E18.5 (Figure 5J; Figure S7D, Supporting Information). However, the enrichment of these pathways was more pronounced at E18.5, with a larger number of DEGs and stronger statistical significance (Figure 5J; Figure S7D, Supporting Information). Moreover, the majority of top‐upregulated genes were canonical ISGs at E18.5 (Figure 5K; Figure S7E, Supporting Information). Consistent with the RNA‐seq data, the fold change in the expression of representative ISGs (*Oasl1a*, *Ifitm3*, *Irf7*, and *Isg‐15*) between the *Adar1^PKO^Ifnar1^‐/‐^* and *Adar1^f/f^Ifnar1^‐/‐^* placentas was elevated at E18.5 compared to E14.5 (Figure 5L; Figure S7F, Supporting Information), while it remained lower than those identified in *Adar1^PKO^* placenta at E14.5 (Figure 1). These data suggest that other MDA5/MAVS‐dependent cytokine signaling pathways (i.e., pro‐inflammatory cytokines (Il‐6, Il‐1β, and Tnf‐α) and type III IFN) parallel to type I IFN,^[^ ^29^ ^]^ may contribute to the FGR phenotype of *Adar1^PKO^Ifnar1^‐/‐^* mice. To this further, we assessed the relative expression of proinflammatory cytokines and found that *Ifnl*, but not *Il‐6*, *Il‐1β*, and *Tnf‐α*, were significantly increased in the *Adar1^PKO^Ifnar1^‐/‐^* placenta (Figure 5L). Additionally, ISH results showed that *Isg‐15* and *Zbp1* are specifically and highly expressed in the JZ region of *Adar1^PKO^Ifnar1^‐/‐^* placentas (Figure 5N). Given that type III IFN induces ISG expressions similar to type I IFN, these results suggest the expression of IFN‐λ enhanced by *Adar1* deficiency may drive the alternative expression of a set of ISGs (Figure 5L‐N; Figure S7F, Supporting Information), albeit less potent than type I IFNs,^[^ ^30^ ^]^ which led to FGR rather than embryonic lethality.

Consistent with the growth retardation of *Adar1^PKO^Ifnar1^‐/‐^* mice, the functions of *Adar1^PKO^Ifnar1^‐/‐^* placentas were compromised at E18.5, as shown by GO terms including secretion, female pregnancy, and reproductive process (Figure 5O), though these defects were not evident at E14.5. This was further confirmed by the dramatically reduced expression of PSGs as pregnancy proceeded (Figure 5P; Figure S7G,H, Supporting Information). In summary, *Ifnar1* deletion fully rescues the embryonic lethality of *Adar1^PKO^* mice but leaves residual FGR, which may be attributed to a non‐redundant Type III IFN response in the placenta.

### 2.6 *Pkr* Knockout Completely Prevents Embryonic Mortality of *Adar1^PKO^* Mice

**Figure 6:**
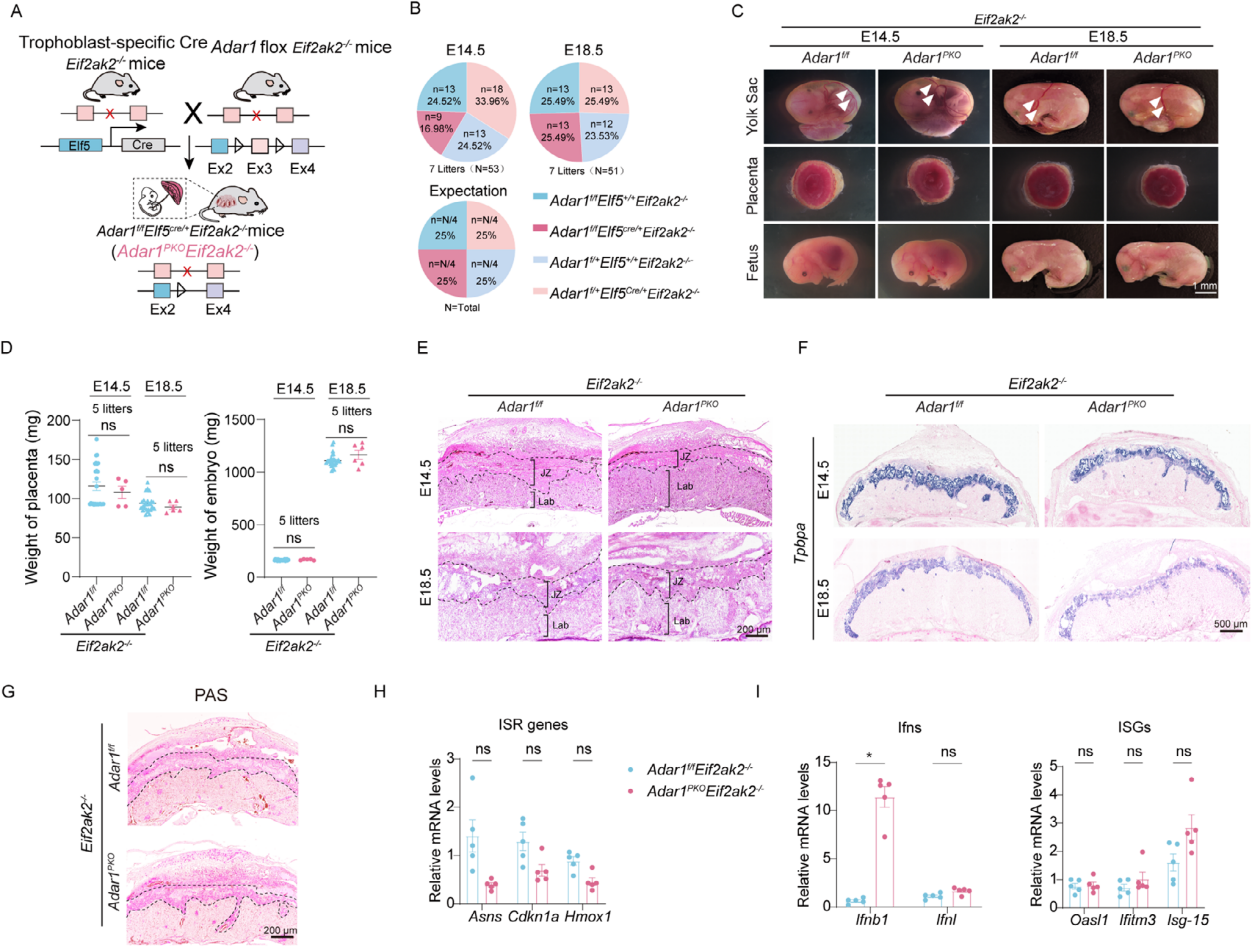
*Pkr* deficiency completely rescues embryonic mortality of *Adar1^PKO^* mice. A) The schematic diagram illustrates the mating strategy to generate trophoblast‐specific *Adar1* deletion on the *Eif2ak2^‐/‐^* background. B) Genotypic distribution of embryos at E14.5, and E18.5 from *Adar1^f/f^ Eif2ak2^‐/‐^* dams mated to *Adar1^f/+^Elf5^Cre/+^Eif2ak2^‐/‐^* sires. C) Stereomicroscopic images of the yolk sac, placenta, and fetus from *Adar1^f/f^Eif2ak2^‐/‐^* and *Adar1^PKO^Eif2ak2^‐/‐^* embryos at E14.5 and E18.5. Arrowhead, yolk sac vasculature. D) Placental and embryonic weights of *Adar1^f/f^Eif2ak2^‐/‐^* and *Adar1^PKO^Eif2ak2^‐/‐^* embryos at E14.5 and E18.5. Dots represent individual mice. E,F) H&E staining E) and *Tpbpa* ISH F) of the placenta from *Adar1^f/f^Eif2ak2^‐/‐^* and *Adar1^PKO^Eif2ak2^‐/‐^* embryos at E14.5 and E18.5. G) PAS staining of placental sections from *Adar1^f/f^Eif2ak2^‐/‐^* and *Adar1^PKO^Eif2ak2^‐/‐^* embryos at E14.5. H,I) RT–qPCR analysis of relative mRNA expression of ISR (H), IFNs, and ISG genes I) from *Adar1^f/f^Eif2ak2^‐/‐^* and *Adar1^PKO^Eif2ak2^‐/‐^* placental tissues at E14.5 (n = 5, 5 litters). In panels D, H, and I, Data are presented as mean ± SEM. *p < 0.05; ns, non‐significant; unpaired *t* test was used.

The expression of *Eif2ak2* (Pkr) is specifically elevated in IJZP (Figure 2D,F), prompting further investigation into its function. Although it has been previously suggested that *Eif2ak2* deletion in *Adar1* mutants failed to rescue embryonic lethality,^[^ ^31^ ^]^ subsequent studies indicated that PKR acted as an essential downstream effector of the MDA5‐dependent IFN response^[^ ^19^, ^32^ ^]^ or as a dsRNA sensor parallel to MDA5 in different contexts,^[^ ^33^ ^]^ which are vital for the early postnatal death. Thus far, the specific contribution of PKR to embryonic lethality has not been thoroughly studied. To address this, we intercrossed *Adar1^f/f^* mice with *Elf5‐Cre* mice on an *Eif2ak2^‐/‐^* background (referred to as *Adar1^PKO^Eif2ak2^‐/‐^*) to evaluate PKR's contribution to placental dysfunctions in *Adar1^PKO^* mice (**Figure** **6**A). Remarkably, *Adar1^PKO^Eif2ak2^‐/‐^* fetuses were completely rescued to birth, accompanied by improved placenta and embryo weight (Figure 6B–D). Furthermore, the structural defects of the *Adar1^PKO^* placenta were restored by *Eif2ak2* deletion (Figure 6E). In addition, the ISH signals of *Tpbpa* were comparable to controls (Figure 6F), in line with the recovered function of glycogen storage in the *Adar1^PKO^Eif2ak2^‐/‐^* placenta (Figure 6G). In agreement with these observations, the expression of three ISR genes, markers indicating PKR activation, was comparable to controls (Figure 6H), suggesting that the ISR in *Adar1^PKO^* placentas depends on PKR. However, except for the expression of *Ifnb1*, *Ifnl2* and the ISGs in the *Adar1^PKO^Eif2ak2^‐/‐^* placenta appeared barely distinguishable from those in the *Adar1^f/f^Eif2ak2^‐/‐^* placenta, contrary to a previous finding that the ISG expression remained significantly elevated in *Adar1^P195A/p150‐^Eif2ak2^‐/‐^* mice (Figure 6I).^[^ ^32^ ^]^ The molecular mechanisms underlying this disparity remain to be elucidated. The similar rescued phenotypes in *Adar1^PKO^Eif2ak2^‐/‐^* and *Adar1^PKO^Mavs^‐/‐^* mice suggest that PKR serves as an essential downstream effector of the MDA5‐dependent IFN response in the context of *Adar1*‐specific deletion in the placenta.

### 2.7 eIF2B Activator Protects Fetal Development from JZ Defects Caused by Placenta‐Specific *Adar1* Deficiency

Given that *Pkr* knockout fully rescued embryonic mortality in *Adar1^PKO^* mice, we further investigated the detailed PKR‐dependent mechanisms causing placental dysfunctions. PKR has been reported to regulate cell death pathways, including Casp‐8‐dependent apoptosis and Casp‐1‐mediated pyroptosis.^[^ ^34^ ^]^ Furthermore, the disappearance of terminally differentiated JZ cells upon *Adar1* deletion (Figure 2) prompted us to evaluate whether cell death pathways directed by Pkr contributed to the *Adar1^PKO^* phenotypes. To this end, we respectively intercrossed *Adar1^f/f^* mice with *Elf5‐Cre* mice on the *Ripk3^‐/‐^*, *Casp‐8^‐/‐^Ripk3^‐/‐^* or *Casp‐1^‐/‐^* background to generate *Adar1^PKO^Ripk3^‐/‐^*, *Adar1^PKO^Ripk3^‐/‐^Casp‐8^‐/‐^* or *Adar1^PKO^Casp‐1^‐/‐^* mice. Unexpectedly, although the placenta defects of *Adar1^PKO^Ripk3^‐/‐^* and *Adar1^PKO^Ripk3^‐/‐^Casp‐8^‐/‐^* mice were partially ameliorated compared to *Adar1^PKO^Casp‐1^‐/‐^* placentas, their corresponding fetus still died around E14.5. They also displayed reduced placental and embryonic weights, with concomitant disrupted placental structure (Figure S8, Supporting Information), indicating that blocking cell death failed to rescue PKR‐mediated JZ defects in the *Adar1^PKO^* placenta.

**Figure 7:**
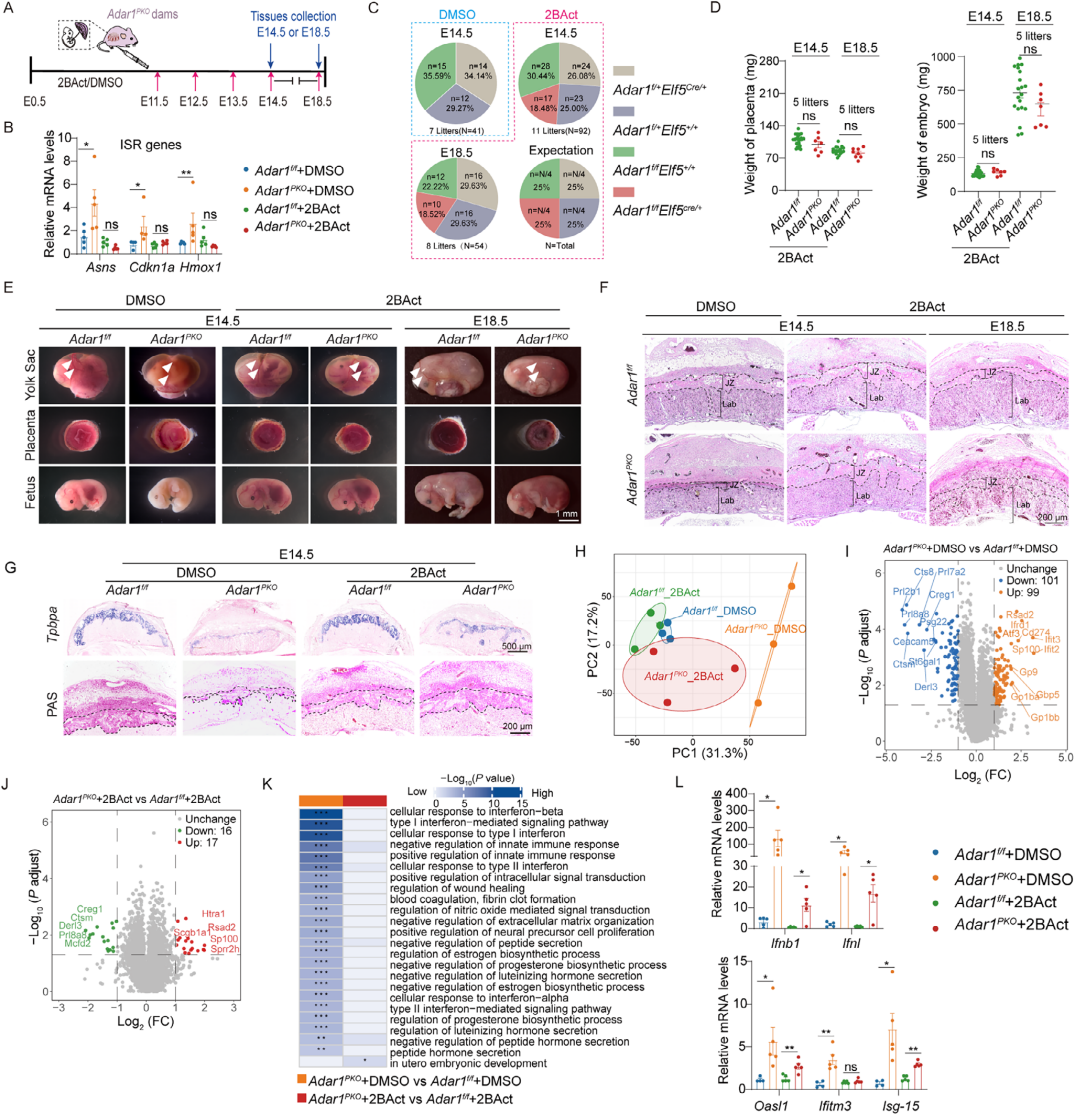
eIF2B activator protects fetal development from JZ defects caused by *Adar1* placenta‐specific deficiency. A) Schematic showing the timeline for 2BAct (intraperitoneal daily injection,1.0 mg kg^−1^) or DMSO treatment from E11.5 to E14.5 or E18.5. B) RT–qPCR analysis of ISR mRNA expression from *Adar1^f/f^* and *Adar1^PKO^* placentas at E14.5 treated with 2BAct or DMSO (n = 5, 5 litters). C) Genotypic distribution of embryos at E14.5, and E18.5 from *Adar1^f/f^* dams mated to *Adar1^f/+^Elf5^Cre/+^* sires and treated with 2BAct or DMSO. D) Placental and embryonic weights of *Adar1^PKO^* dams treated with 2BAct at E14.5 and E18.5. Dots represent individual mice. E) Stereomicroscopic images of the yolk sac, placenta, and fetus from *Adar1^PKO^* dams treated with 2BAct or DMSO. Arrowheads, yolk sac vasculature. F,G) H&E staining F), *Tpbpa* ISH, and PAS staining G) of placental sections from *Adar1^PKO^* dams treated with 2BAct or DMSO at E14.5 and E18.5. H) PCA of the proteomic profiles of *Adar1^f/f^* and *Adar1^PKO^* placentas (3 litters, n = 3) treated with 2BAct or DMSO. I,J) Volcano plot of proteomic data showing differentially expressed proteins in *Adar1^f/f^* and *Adar1^PKO^* placentas upon DMSO I) or 2BAct J) treatment. K) GO analysis of differentially expressed proteins in *Adar1^f/f^* and *Adar1^PKO^* placentas treated with 2BAct or DMSO. L) RT–qPCR analysis of IFNs and ISGs expression (n = 5, 5 litters). In panels B, D, and L, Data are presented as mean ± SEM. *p < 0.05, **p < 0.01; ns, non‐significant; unpaired *t* test was used.

In addition to cell death, ISR is another key downstream player activated by PKR. As one of four classical ISR sensor kinases, activated PKR in response to dsRNA accumulation phosphorylates eIF2α, causing a shutdown of translation initiation and a selective expression of ISR genes.^[^ ^35^ ^]^ Thus, we reasoned that self‐RNA unsequestered by ADAR1 would trigger PKR activation, thereby inducing ISR in the *Adar1^PKO^* placenta. As anticipated, the expression of the ISR genes (*Asns, Cdkn1a*, and *Hmox1*) was significantly elevated in the *Adar1^PKO^* placenta (Figure S9A, Supporting Information). Subsequently, we tested whether the ISR inhibitor 2BAct could alleviate the pathology of the *Adar1^PKO^* placenta. *Adar1^PKO^* or *Adar1^f/f^* mice were subjected to daily intraperitoneal injection of DMSO or 2BAct from E11.5 (before the initiation of IFN activation) (Figure S9B, Supporting Information) to E14.5 or E18.5 (**Figure** **7**A). RT‐qPCR revealed that mRNA expression of representative ISR genes in 2BAct‐treated *Adar1^PKO^* placentas was restored to control levels, validating the efficacy of in vivo 2BAct treatment (Figure 7B). Notably, 2BAct treatment nearly completely prevented the fetal mortality of *Adar1^PKO^* mice, without causing maternal hepatorenal toxicity (Figure 7C; Figure S9C, Supporting Information), and significantly mitigated weight loss of *Adar1^PKO^* fetuses and placentas at E14.5 (Figure 7D). Simultaneously, the gross morphology of the placenta, fetus, and yolk sac was indistinguishable from the DMSO‐treated *Adar1^f/f^* mice (Figure 7E). In addition, prominently rescued JZ thickness, *Tpbpa* expression, and glycogen storage, along with partially rescued PSGs and Ceacams expression were observed in the *Adar1^PKO^* placenta following 2BAct treatment (Figure 7F,G; Figure S9D,E Supporting Information), suggesting that structural integrity and functional defects of the *Adar1^PKO^* placenta can be restored upon ISR blockage in vivo.

To further characterize the translational reprogramming fine‐tuned by the PKR‐eIF2a axis specifically in the placenta, we performed mass spectrometry (MS)‐based proteomics to monitor proteomic changes across the four experimental groups at E14.5. PCA of the proteomics results showed that the protein expression profiles of placentas from 2BAct‐treated *Adar1^PKO^* mice closely clustered with those from *Adar1^f/f^* mice treated with 2BAct or DMSO, while apparently segregated from *Adar1^PKO^* mice injected with DMSO (Figure 7H). Remarkably, unlike the DMSO*‐Adar1^PKO^* mice (Figure 7I), most of the upregulated and downregulated proteins were restored to normal levels in 2BAct‐treated *Adar1^PKO^* placentas (Figure 7J). Protein‐protein Interaction network analysis on these differentially expressed proteins revealed that the clustering of ISG proteins was enriched in DMSO‐injected *Adar1^PKO^* placentas, a feature that was abolished in 2BAct‐treated *Adar1^PKO^* placentas (Figure S9F, Supporting Information). GO analysis of differentially changed proteins showed that subcategory terms related to IFN response, including cellular response to interferon‐beta, type I interferon‐mediated signaling pathway, cellular response to type I interferon, etc., were enriched in DMSO‐treated *Adar1^PKO^* placenta but returned to the baseline in placentas treated with 2BAct (Figure 7K). Furthermore, the mRNA expression of ISGs remained modestly elevated in 2BAct‐treated *Adar1^PKO^* placenta at E14.5, further verifying that 2BAct treatment suppressed the IFN response induced by *Adar1* ablation (Figure 7L; Figure S9G, Supporting Information). Collectively, these results suggest that the embryonic lethality of *Adar1^PKO^* mice is a primary consequence of placental ISR and occurs independently of cell death, which could be significantly alleviated by 2BAct treatment.

## 3 Discussion

Here, we demonstrate that selective *Adar1* deletion in the placenta disrupts the homeostatic balance of intrinsic IFN response within the JZ, which fatefully contributes to embryonic lethality. Mechanistically, fetal demise is induced by the dsRNA‐MAVS‐IFN‐PKR‐ISR signaling axis, but not ZBP1 or cell death effectors (i.e., Caspase‐8, Caspase‐1, and Ripk3). For the first time, we define unique features of the endogenous placental IFN response to *Adar1* deficiency, including its spatiotemporal heterogeneity, the impact on IJZP‐to‐JZ trophoblast differentiation, and the origin of trigger dsRNAs from the 3′ UTR of ISG mRNAs. Notably, ISR inhibition with small‐molecule 2BAct mitigated JZ defects in *Adar1^PKO^* placentas and prevented the associated embryonic lethality (Figure S10, Supporting Information), representing a novel therapeutic strategy for precision treatments of pregnancy complications linked to interferonopathies while preserving other antiviral responses.

Our study suggests that the IFN responses and placental disruptions were predominantly confined to JZ in *Adar1^PKO^* mice. These findings highlight the heterogeneity of endogenous IFN response across different murine trophoblast cell types. In this study, we leveraged snRNA‐seq and identified a new trophoblast subtype in *Adar1^PKO^* placentas, IJZP, which displayed elevated IFN pathway activity and robust IFN response signatures. As a progenitor cell, IJZP could differentiate into the trajectories of GlyT and SpT, and should not accumulate in large numbers at E14.0.^[^ ^36^ ^]^ Although the mechanisms of impaired IJZP differentiation in the *Adar1^PKO^* placenta need to be investigated, our data imply that IJZP with excessive IFN response fails to properly differentiate into JZ trophoblast lineages (GlyT I, GlyT II, and SpT), which may be the initial cause of the impaired JZ morphogenesis in the *Adar1^PKO^* placenta. Concordantly, the prominent functions of JZ, including glycogen storage and the secretion of pregnancy‐associated proteins,^[^ ^21^ ^]^ were compromised in the *Adar1^PKO^* placenta. Glycogen storage acts as an energy reserve to provide the necessary nutrition to the fetus, particularly during the high‐demand periods of fetal growth.^[^ ^37^ ^]^ Thus, aberrant placental glycogen storage in the *Adar1^PKO^* placenta may contribute to FGR and compromise fetal survival. Furthermore, the expression of pregnancy‐associated proteins, especially PSGs, was specifically downregulated in the context of placental IFN hyper‐response in both *Adar1^PKO^* mice and humans, highlighting its potential as a diagnostic biomarker for pregnancy disorders caused by IFN. Collectively, our findings delineate IFN response heterogeneity across different trophoblast subtypes and offer new insights into cellular characteristics of placental pathology linked to interferonopathies at the maternal‐fetal interface.

*Adar1* deficiency induces a strong IFN response due to the accumulation of dsRNA or Z‐RNA, which is recognized by MDA5‐MAVS or ZBP1, respectively.^[^ ^23^, ^38^ ^]^ Our data from *Adar1^PKO^Mavs^‐/‐^* or *Adar1^PKO^Zbp1^‐/‐^* mice confirmed that *Mavs* deficiency completely rescued *Adar1^PKO^* mice from embryonic lethality, restored their placental defects, and prevented the IFN production and ISG signatures in the placenta, contrary to the phenotypes of *Adar1^PKO^Zbp1^‐/‐^* mice. These results prove that MAVS, not ZBP1, is the specific dsRNA effector responsible for the detrimental IFN response in the *Adar1^PKO^* placenta. Additionally, ZBP1‐mediated apoptosis and necroptosis are dispensable for the pathology of the *Adar1^PKO^* placenta, suggesting that the contribution of ZBP1 to embryonic lethality in this model might be subtle. These results are consistent with a recent study showing that concurrent deletion of *Zbp1* delayed fetal lethality only by one gestational day, despite its major contribution to the postnatal mortality of *Adar1^mZα/–^* mice via apoptosis and necroptosis.^[^ ^39^ ^]^ Taken together, the role of ZBP1 varies across different *Adar1* mutant models, which needs to be thoroughly explored.

Our findings demonstrate that placental trophoblasts constitutively expressed Type I IFN and ISGs, in agreement with a recent study showing that self‐SINEs‐derived dsRNA induces viral mimicry response to trigger placental Type III IFN expression during normal pregnancy.^[^ ^10^ ^]^ Of note, the peak of constitutive ISG expression under physiological conditions coincides with the embryonic death of *Adar1^PKO^* mice, which occurs approximately at E14.5. This phenomenon could be attributed to the accumulation of ISGs‐derived dsRNAs by positive feedback, as our results demonstrate. Specifically, 3′ UTRs of ISG mRNAs that harbor SINE repetitive elements could fold into immunogenic dsRNAs in the *Adar1^PKO^* placenta, similar to the scenario of Z‐RNAs formation from ISG mRNAs.^[^ ^27^ ^]^ Thus, we propose that some *Adar1* deficiency‐induced ISGs give rise to “naked” SINEs/Alu dsRNAs, forming a major class of MDA5 substrates and amplifying the spark of initial IFN signaling to a much greater magnitude through positive feedback regulation. Intriguingly, we did not note significant interferonopathies in *Adar1^PKO^* mice until E14.5, even though *Adar1* deletion driven by *Elf5‐Cre* was initiated as early as the blastocyst stage. This suggests the constitutively high level of IFN response during mid‐to‐late pregnancy mounts the activity of the dsRNA‐MDA5‐IFN‐ISG‐dsRNA feedback loop to a level beyond what the placenta can tolerate. Thus, ADAR1, evolved as an ISG, acts as a placental innate immunity rheostat to cut the risk of a viral mimicry response that constitutively induces IFNs, thereby conferring antiviral protection in trophoblasts without causing embryonic lethality.

Our study also highlights the importance of the ISG remnants in *Adar1^PKO^* mice with concurrent *Ifnar1* deletion. Although a previous study has demonstrated that the loss of *Ifnar1* in *Adar1* ^‐/‐^ mice still results in fetal death at E15.5,^[^ ^16^ ^]^ we showed that removal of *Ifnar1* completely rescued the embryonic lethality of *Adar1^PKO^* mice, similar to that concurrent deletion of *Mavs* rescues the *Adar1^PKO^* fetuses to birth. Of note, the *Adar1^PKO^Ifnar1^‐/‐^* fetuses displayed FGR at E16.5‐E18.5, and the ISG signatures in the corresponding placenta were also significantly enhanced as the pregnancy progressed. Emerging evidence has shown that *Adar1* and *Ifnar1* double‐mutant embryos still maintain high levels of ISGs independently of type II IFN.^[^ ^16^, ^40^ ^]^ These results suggest that other MDA5/MAVS‐dependent cytokine signaling, such as IFN‐λ, Il‐6, Il‐1β, and Tnf‐α,^[^ ^29^ ^]^ are important for the FGR phenotype. Among these cytokines, relative *Ifnl2* expression was significantly increased in the *Adar1^PKO^Ifnar1^‐/‐^* placenta, accompanied by a pronounced induction of ISG‐specific expression in the progressively diminished JZ as the pregnancy progressed beyond E14.5. Given that differences in signaling magnitude, kinetics, and the cell types that respond to type I and type III IFNs result in different biological outcomes,^[^ ^30^ ^]^ further investigation is warranted to determine whether the enhanced expression of IFN‐λ associated with *Adar1* deficiency in the placenta, in parallel to type I IFN, promotes the alternative expression of a set of ISGs causing FGR.

To establish a formidable barrier restricting transplacental viral transmission, the placenta spontaneously produces type III IFN through constitutively active MDA5 and PKR, which recognize self‐SINEs‐derived dsRNAs from imprinted microRNA clusters under physiological conditions. However, the trade‐off of this antiviral mechanism is that MDA5 and PKR may inappropriately recognize other structurally similar self‐SINEs‐derived dsRNAs, potentially triggering an uncontrolled and exaggerated IFN response. Analogous to *Adar1^PKO^Mavs^‐/‐^* mice, healthy *Adar1^PKO^Eif2ak2^‐/‐^* pups were born alive, demonstrating that PKR serves as a key downstream effector of IFN‐triggered adverse pregnancy outcomes. Taken together, our results showed that the baseline activity of constitutive MDA5 and PKR in placentas was amplified by accumulated self‐dsRNA in *Adar1^PKO^* mice, thereby leading to uncontrolled IFN production and placenta injury. This indicates that the endogenous dsRNAs for MDA5 or PKR activation should be part of ADAR1's substrates. Coincidentally, emerging evidence shows that IFN promotes PKR activation in *Adar1* KO cells because of defective ADAR1 competitive binding for PKR substrate dsRNAs that originate from a subset of mRNAs.^[^ ^19^, ^33^ ^]^ This notion is similar to our finding that ISG mRNAs were efficiently pulled down by MDA5 in the *Adar1^PKO^* placenta. Therefore, these observations suggest that PKR and MDA5 may share some mRNA‐derived dsRNA substrates induced by IFN. Contrary to a previous finding that the ISG expression remained significantly elevated upon *Eif2ak2* deletion in *Adar^P195A/p150‐^* offspring organs,^[^ ^32^ ^]^ we demonstrated that the elevated *IFN* and *ISG* expression in *Adar1^PKO^Eif2ak2^‐/‐^* placentas were prominently reduced to the level in *Adar1^f/f^Eif2ak2^‐/‐^* placentas, indicating an unknown placenta‐specific mechanism underpinning PKR‐driven IFN production and response. In summary, RNA modifier ADAR1 acts as a placental innate immunity rheostat by reducing the dsRNA burden of constitutively active PKR and MDA5 and the corresponding risk of overwhelming IFN responses causing placenta damage.

PKR, ZBP1, and MAVS have been reported to regulate cell death pathways, including Casp‐8‐dependent apoptosis, Mlkl‐dependent necroptosis, and Casp‐1‐mediated pyroptosis.^[^ ^34^, ^41^, ^42^ ^]^ However, our work indicates a minimal contribution of Casp‐8, Ripk3, and Casp‐1 to the *Adar1^PKO^* placental defects, suggesting that these death pathways in the *Adar1^PKO^* placenta are not as important as emphasized in *Adar1^Zα/–^* and *Adar1^P195A/p150null^*.^[^ ^41^ ^]^ Conversely, our study sheds light on the importance of ISR, another significant downstream pathway activated by PKR,^[^ ^43^ ^]^ in the context of placental *Adar1* deficiency. We demonstrated that the ISR inhibitor 2BAct protects fetal development from JZ defects caused by *Adar1* placenta‐specific deletion. Consistent with our work, a recent study indicated that 2BAct can rescue intrauterine neurodevelopmental defects without reproductive toxicity,^[^ ^44^ ^]^ suggesting its potential as a pregnancy‐targeted ISR drug. Our study has important implications regarding the treatment and understanding of pregnancy disorders. For the first time, our study links ISR with placental injury and malfunctions caused by excessive IFN response. In addition to our findings, compelling pieces of evidence have repeatedly shown that both “TORCH” infections and genetic interferonopathies with high IFN levels are strongly associated with adverse pregnancy outcomes.^[^ ^4^, ^6^, ^7^, ^8^ ^]^ However, blocking IFN may be an ill‐advised strategy to mitigate pregnancy complications linked to interferonopathies, since it would indiscriminately suppress constitutively expressed ISGs in the placenta, potentially rendering the conceptus susceptible to viral invasion. Here, we identified 2BAct‐mediated ISR inhibition as a potential therapeutic strategy for treating these pregnancy disorders by targeting a specific IFN downstream effector while maintaining other antiviral responses, although achieving clinical transformation requires more comprehensive studies. In addition to interferonopathies, an earlier study has reported that patients with eIF2B‐related disorders displayed serious encephalopathy, oligohydramnios, and FGR during early embryonic development,^[^ ^45^ ^]^ but the pathogenic mechanism remains unknown. We anticipate that our work unmasks a common pathogenic mechanism for pregnancy complications associated with eIF2B‐related disorders. More broadly, we propose that the connection between ISR activation and pregnancy complications might extend to conditions where the ISR is induced by various triggers other than IFN activation.^[^ ^43^ ^]^

Collectively, our study elucidates that ADAR1 is indispensable for fine‐tuning spatially restricted intrinsic IFN‐mediated ISR positively amplified by ISG‐3′UTR‐dsRNA in the mouse placenta, thereby modulating the balance between embryonic development and dsRNA‐induced antiviral defense at the maternal‐fetal interface. Our findings provide proof of concept for a therapeutic strategy targeting the ISR to mitigate pregnancy complications linked to interferonopathies.

## 4 Experimental Section

### Mice

All mice used in this study were bred and maintained on a C57BL/6J background. *Elf5‐Cre* mice which drive Cre‐mediated recombination in trophoblast cells were generously provided by Dr. Haibin Wang at Xiamen University.^[^ ^28^ ^]^ *Adar1^f/f^*, *Mavs^‐/‐^*, and *Eif2ak2^‐/‐^* mice were purchased from GemPharmatech Co., Ltd. *Ifnar1^‐/‐^*, *Zbp1^‐/‐^*, *Ripk3^‐/‐^*, *Casp8^‐/‐^*, and *Casp1^‐/‐^* mice were generated and kindly provided by Dr. Jiahuai Han at Xiamen University.^[^ ^46^, ^47^ ^]^

All mice were housed in a pathogen‐free environment and had free access to water and food. Breeding and experimental procedures were conducted with prior approval of the Animal Care and Use Committee of Xiamen University (XMULAC20210168). Dams and sires aged between 8 and 12 weeks were cohoused, and embryonic day 0.5 (E0.5) of pregnancy was defined as the first observation of a vaginal plug. The embryos and corresponding placentas were dissected, weighed, and imaged using a Nikon SMZ18 stereo microscope. The primers for genotyping PCR are listed in Table S1 (Supporting Information).

### RNA Extraction and RT‐qPCR

Total RNA was extracted from mouse tissues using TRIzol reagent (Invitrogen, 15 596 018) following the manufacturer's protocol. To synthesize cDNA, 0.5 µg of total RNA was reverse transcribed using the PrimeScript RT reagent Kit with gDNA Eraser (TAKARA, RR047A). Real‐time PCR was performed on a QuantStudio 5 Real‐Time PCR system (Life Technologies) with qPCR Mix (2 ×) (Vazyme, Q712‐03). Data analysis was performed using the 2^‐ΔΔCt^ method after normalization to the Gapdh internal control. The RT‐qPCR primers are listed in Table S2 (Supporting Information).

### Protein Extraction and Immunoblotting

Frozen mouse placentas were ground in ice‐cold RIPA lysis buffer. After centrifugation, protein lysates were separated by SDS‐PAGE and transferred to methanol‐activated polyvinylidene difluoride (PVDF) membranes. The membranes were then blocked with 5% skim milk in TBST for 1 h at room temperature and incubated overnight at 4 °C with a primary antibody: anti‐ADAR1 (Abclonal, A7869, 1:1000) and anti‐actin (CST, 8457S, 1:1000). After incubation with secondary antibodies (NeoBioscie, ANR02‐2, 1:5000), film exposure was performed and visualized using Supersignal West Pico (Thermo Scientific).

### In Situ Hybridization (ISH)

ISH was performed as previously described.^[^ ^48^ ^]^ Briefly, frozen sections (10 µm) were dried at 37 °C for 5 min and then fixed in 4% paraformaldehyde (PFA) for 1 h at room temperature. Sections were hybridized with specific digoxigenin‐labeled RNA probes overnight at 60 °C. Nonspecific hybridization was removed by RNase A digestion (SIGMA, R4875‐100, 10 mg mL^−1^) at 37 °C for 30 min. The sections were then blocked and incubated overnight at 4 °C with alkaline phosphatase‐conjugated sheep anti‐digoxigenin Fab fragments (Roche, 11 093 274 910, 1:3000). Probe‐cRNA hybrids were detected using NBT/BCIP (Sangon Biotech, C520019), followed by nuclear counterstaining with Nuclear Fast Red (Solarbio, G1321). After washing, the sections were cleared in xylene and mounted with a mounting medium for subsequent imaging procedures. The primers used to make specific mouse RNA probes labeled with digoxin or fluorescein are listed in Table S3 (Supporting Information).

### Single‐Cell Resolution In Situ Hybridization on Tissues (SCRINSHOT)

The padlock probes were designed following the pipeline described previously.^[^ ^49^ ^]^ Briefly, the sequences for the 5′ and 3′ arms of the padlock probes, which are complementary to the target mRNA, were generated using the PrimerQuest Tool. The Padlock Design Assistant (https://github.com/AlexSount/SCRINSHOT) was then used to finalize the design of the padlock probes.

Frozen tissue sections were fixed in 4% PFA in PBS for 1 h at room temperature. After prehybridization, the placental tissue sections were hybridized with specific padlock probes at 45 °C for 2 h. Post‐hybridization, the slides were incubated at 37 °C for 12–16 h to facilitate ligation of the hybridized padlock probes using SplintR ligase (NEB, M0375L). Subsequently, rolling circle amplification was performed by incubating the slides in phi29 DNA polymerase (NEB, M0269L) buffer at 37 °C for 12–16 h. The gene‐specific domain of the padlock probes was then detected using FITC‐Tyramide (Servicebio, G1223). The specific padlock probes utilized are listed in Table S4 (Supporting Information).

### Dot Blot

Equal amounts of RNA from mouse placentas were dropped onto a nitrocellulose membrane. The membrane was then subjected to ultraviolet crosslinking and blocked with 5% skim milk for 1 h at room temperature. After blocking, the membrane was immunoblotted overnight at 4 °C with J2 mouse monoclonal anti‐dsRNA antibody (SCICONS, 10 010 500, 1:5000). Following immunoblotting, the membrane was incubated with the corresponding secondary antibody (NeoBioscie, ANM02‐1, 1:5000) for 1 h at room temperature. The results were visualized using Supersignal West Pico (Thermo Scientific) and methylene blue was stained as a loading control.

### Immunofluorescence (IF)

The frozen tissue sections were fixed in 4% PFA for 15 min and washed three times with ice‐cold PBS. Subsequently, the sections were permeabilized in PBS containing 0.1% Triton X‐100 for 10 min and blocked in PBS containing 1% BSA (Sigma, A9418‐100G) and 10% goat serum (Solarbio, SL038‐10m) for 1 h. The blocked sections were then incubated with the indicated primary antibodies overnight at 4 °C before incubation with the secondary antibody and DAPI staining. All images were captured using a Zeiss LSM 880+Airyscan and processed with Zeiss ZEN. The following primary antibodies and dilutions were used: J2 anti‐dsRNA antibody (SCICONS, 10 010 500, 1:200), anti‐Mct1 (Sigma‐Aldrich, #AB1286‐I, 1:500), anti‐Mct4 (Sigma‐Aldrich, #AB3314P, 1:500). Two secondary antibodies were used: Alexa Fluor 488‐labeled goat anti‐mouse and Alexa Fluor 594‐labeled goat anti‐mouse (Jackson ImmunoResearch, 1:400).

### Histological Analysis and Immunohistochemistry (IHC)

Fresh mouse tissues were fixed in 4% PFA, dehydrated, paraffin‐embedded, and cut into 5‐mm sections. The deparaffinized sections were used for the hematoxylin and eosin (H&E) staining, periodic acid‐Schiff (PAS) staining, and IHC analysis. For H&E staining, the rehydrated slides were stained using a Hematoxylin‐Eosin Stain kit (Solarbio, #G1120) following the manufacturer's protocol. All slides were mounted with a resin mounting medium (Solarbio, JY123). For PAS staining, slides were treated with 1% (wt./vol) periodic acid (Solarbio, G1280) in distilled H_2_O_2_ for 5 min at room temperature. Slides were washed in running water, incubated in Schiff's reagent (Solarbio, G1280) for 10 min at room temperature, rinsed in running tap water, counterstained with hematoxylin (Solarbio, #G1120), and mounted. For immunohistochemical analysis, the rehydrated slides were heated in citrate Tris buffer (pH 6.0) in a microwave oven for antigen retrieval. Next, the slides were incubated in 3% H_2_O_2_ to block endogenous peroxidase for 15 min and washed. Sections were blocked in 3% BSA or serum for 1 h and continuously stained by primary antibody Laminin (Sigma‐Aldrich, #L9393, 1:200) and anti‐rabbit secondary antibody (NeoBioscle, ANR02‐2, 1:500). After counterstaining with hematoxylin, sections were mounted with a resin mounting medium (Solarbio, JY123). These histological sections were captured using the Nikon MODEL ECLIPSE Ni‐U microscope.

### Placental Permeability Assay

Pregnant mice received intravenous injections of 200 µL FITC‐labeled dextran (Sigma‐Aldrich, 46945‐100MG‐F, 10 mg mL^−1^) in PBS. After 15 min, the mice were sacrificed, and the corresponding placentas were immediately fixed in 4% PFA for 2 h, followed by snap freezing in liquid nitrogen and embedding in optimal cutting temperature compound. The placentas were then sectioned into 30 µm thick slices and dried at 37 °C for 5 min. After staining with DAPI (Thermo Fisher Scientific, #D1306), the sections were mounted with Fluoromount‐G Anti‐Fade (SouthernBiotech, 0100–35). Images were captured using a Zeiss LSM 880+Airyscan and processed with Zeiss ZEN.

### 2BAct Treatment

Pregnant mice received daily 200 µL intraperitoneal injections of 2BAct (Glpbio, 2143542‐28‐1, 10 mg mL^−1^) in corn oil (Sigma‐Aldrich, C8267) from E11.5 to E14.5 or E18.5. DMSO dissolved in corn oil was utilized as a control. Embryos and placentas of each genotype were harvested for further analysis.

### RNA Sequencing (RNA‐seq) and Analysis

Total RNA from placental tissues (three biological replicates for each group) was extracted using TRIzol reagent (Invitrogen, 15 596 018). RNA‐seq was performed using the MGI‐2000 platform (BGI, China). Paired‐end reads were quality‐filtered using Trim Galore (v0.6.10) and aligned to the mouse genome (mm39) with STAR (v2.7.9a). Gene expression was quantified as FPKM using EdgeR (v4.2.1), and differential expression analysis was conducted with DESeq2 (v1.44.0) (FC > 1.5, FDR < 0.05). Normalized counts were obtained via DESeq2's VST method. GO, KEGG, and GSEA were performed using ClusterProfiler (v4.12.1) and sorted by log_2_FC values. Visualization utilized ComplexHeatmap (v2.20.0) and ggplot2 (v3.5.1) in R (v4.4.1).

### snRNA‐seq and Analysis

Nuclei from *Adar1^f/f^* and *Adar1^PKO^* placental tissues were processed using the Chromium Single Cell 3′ Reagent V3 Kit (10 × Genomics) and sequenced on a NovaSeq 6000 S4 platform. CellRanger (v4.0.0) processed the reads into gene expression matrices. Seurat (v4.3.0) performed quality control, keeping nuclei with >500 genes and <5% mitochondrial content. The top 2000 variable genes were identified, and PCA was used to compute the top 50 components. UMAP was applied for dimensionality reduction and clustering, and nuclei were annotated using known placental markers.^[^ ^36^ ^]^ To delineate JZP2‐I and JZP2‐II, differential expression of ISGs were used, such as *GPB3*, *Eif2ak2*, which were specifically enriched in JZP2‐II. RNA velocity was calculated with the velocyto. R package, with visualization embedded into the UMAP plot.

### RNA Immunoprecipitation (RIP), Sequencing and RT‐qPCR

Placental tissues from E14.5 *Adar1^f/f^* and *Adar1^PKO^* mice were processed to extract the input RNA using TRIzol Reagent and lysed in dsRIP buffer (100 mm NaCl, 50 mm Tris‐HCl pH 7.4, 3 mm MgCl_2_, 0.5% NP‐40) with protease inhibitors and RNase inhibitors. Lysates were incubated with 5 mg MDA5 antibody (Proteintech, 66770‐1‐Ig) or J2 mouse monoclonal anti‐dsRNA antibody (SCICONS, 10 010 500) at 4 °C for 2 h, followed by Protein G Dynabeads capture. RNA captured by beads was extracted and subjected to RT‐qPCR or sequencing. For sequencing, rRNA‐depleted, fragmented, and sequenced on the NovaSeq platform (BGI, China). Reads were processed with FastQC and deduplicated using FastUniq (v1.1). STAR (v2.5.3a) aligned reads to Gencode vM35 transcriptome, and data were normalized using EdgeR's TMM method. TE analysis was performed using GTF files curated by the Hammell Lab (http://labshare.cshl.edu/shares/mhammelllab/www‐data/TEtranscripts/TE_GTF/mm39_rmsk_TE.gtf.gz), and folding energies were calculated with RNAfold from the Vienna RNA package (v2.4.11). The primers used for RIP RT‐qPCR are listed in Table S2 (Supporting Information).

### Proteomics

Proteins from placentas were reduced with 5 mM DTT (30 min, 56 °C), alkylated with 11 mM iodoacetamide (15 min, dark), and digested with trypsin (1:50 ratio, overnight; 1:100 ratio, 4 h). Peptides were desalted and loaded onto a homemade C18 column (25 cm, 100 µm i.d.). The mobile phase was solvent A (0.1% FA, 2% acetonitrile) and solvent B (0.1% FA, 90% acetonitrile). Peptides were separated on an EASY‐nLC 1200 UPLC system (ThermoFisher Scientific) using a gradient of 6–22% B (0–22.5 min), 22–34% B (22.5–26.5 min), 34–80% B (26.5–28.5 min), 80% B (28.5–30 min) at 700 nL min^−1^, and analyzed on an Orbitrap Exploris 480 with nano‐electrospray ion source. FAIMS CV was set to −45 V, with full MS resolution at 60 000 for 350–1400 m/z. MS/MS was conducted with a fixed first mass of 120 m/z and a resolution of 15000, with HCD NCE at 27%. AGC target was 1E6 with a maximum injection time of 22 ms. Peptides were extracted and analyzed by Wuhan Metware Biotechnology Co., Ltd. Data were scaled by unit variance and analyzed via PCA using the prcomp function in R. To avoid overfitting, a permutation test with 200 permutations was performed. Differential proteins between groups were identified using a VIP score >1 and an absolute |log2FC| ≥1.0. GO enrichment analysis on differentially expressed proteins was conducted using the clusterProfiler package (v4.10.1) in R, with significance determined by a p‐value threshold of *p* < 0.05. Protein‐protein interaction (PPI) networks were constructed by searching the differentially expressed protein sequences against the STRING database (v11.0), retaining interactions with a confidence score >0.7. These networks were visualized using the visNetwork package in R.

### Statistics

Data are presented as means ± SEM unless otherwise stated. Statistical significance was assessed using the Student's *t*‐test, performed with GraphPad Prism9 software. Differences were considered to be statistically significant at the *p* < 0.05 (^*^ *p* < 0.05, ^**^ *p* < 0.01, ^***^ *p* < 0.001, ns. non‐significant). The specific number of replicates and experimental conditions for each analysis are detailed in the corresponding figure legends.

## Conflict of Interest

The authors declare no conflict of interest.

## Author Contributions

X.C. and X.X. contributed equally to this work. B.C., X.H., and X.C. designed research; X.C., X.X., J.C., H.Z., H.Z., and W.Z. performed research; X.C., X.X., J.C., H.Z., H.Z., W.Z., and B.C. analyzed and supervised data; B.C., X.H., and W.Y. received research funding supports; X.C., X.X., W. Y., X.H., and B.C. wrote and reviewed the paper.

## Supporting information



Supporting Information

## Data Availability

The data that support the findings of this study are available in the supplementary material of this article.
