# Supplementary material for: ADAR1 as a Placental Innate Immune Rheostat Sustaining the Homeostatic Balance of Intrinsic Interferon Response at the Maternal‐Fetal Interface
Source: Adv Sci (Weinh). 2025 Aug 18;12(42):e05491. doi: 10.1002/advs.202505491 (PMC12622419; doi:10.1002/advs.202505491)
Supplement: Supplementary file 1 — Supporting Information [file ADVS-12-e05491-s001.docx]

**Supplementary Figures**


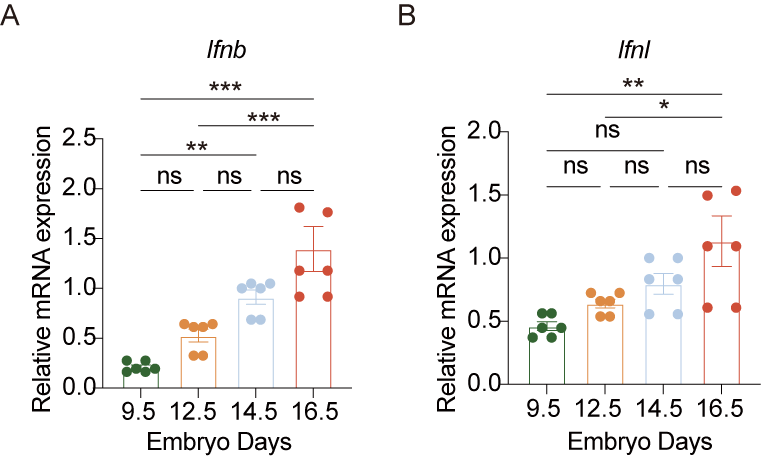


**Figure. S1.** (A-B) RT–qPCR analysis showing the relative expression of IFNs in wild-type placental tissues from E9.5 to E16.5 (n=6, 6 litters). Data are presented as mean ± SEM. **p* < 0.05, ***p* < 0.01, ****p* < 0.001; ns, non-significant; unpaired t test was used.


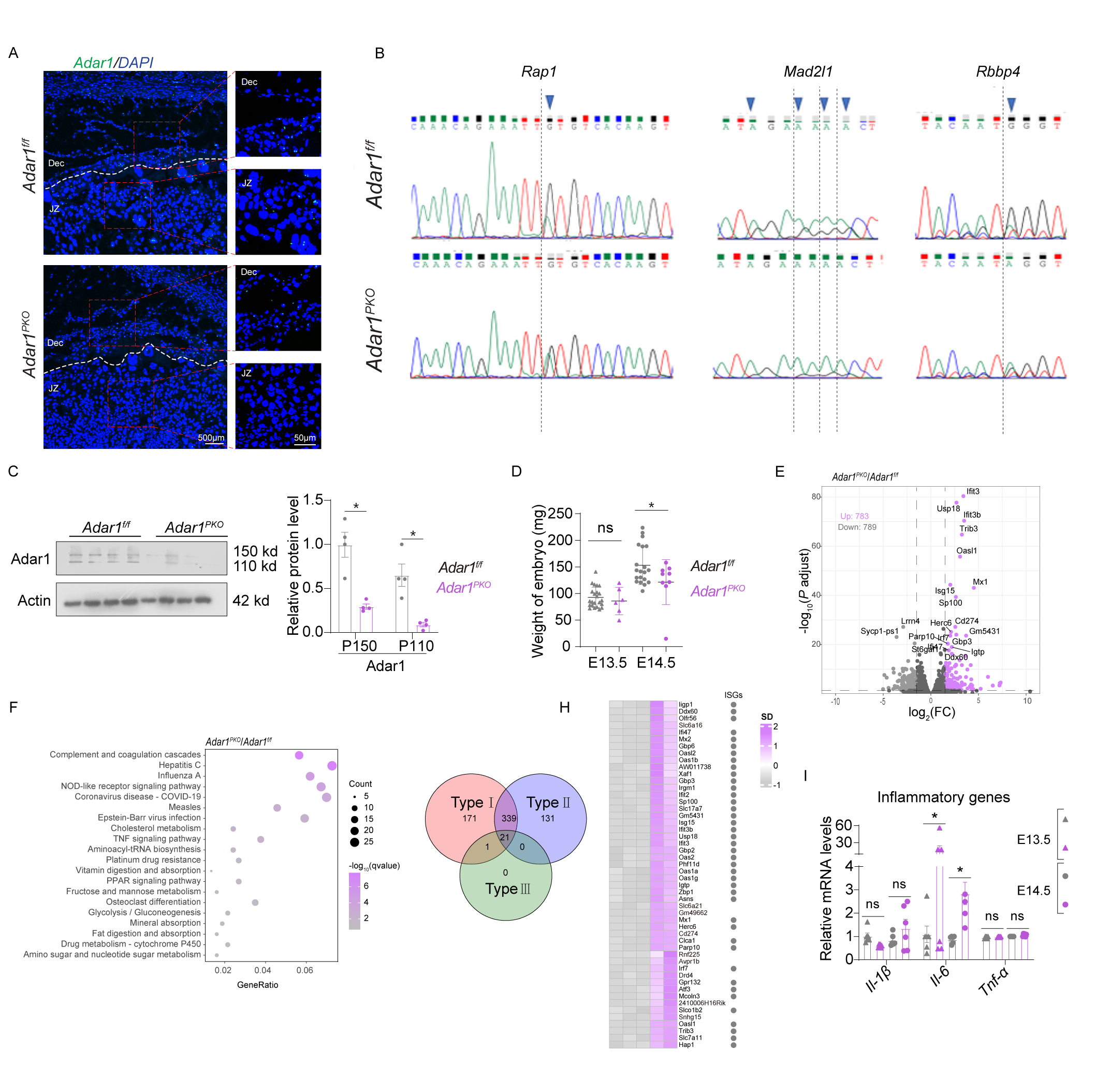


**Figure. S2. *Adar1* deletion in the placenta induced spontaneous IFN responses,** **related to Figure 1**. (A) SCRINSHOT analysis shows *Adar1* expression in decidua (Dec) and JZ (the boundary between them was depicted with the dashed lines) of *Adar1^f/f^* and *Adar1^PKO^* placentas at E13.5. (B) Sanger sequencing traces illustrate site-specific A-to-I RNA editing by *Adar1* in *Rap1*, *Mad2l1*, and *Rbbp4* transcripts. Blue arrowheads, A-to-I editing sites. (C) Western blot of Adar1 in E14.5 *Adar1^f/f^* and *Adar1^PKO^* placentas. Right panel, quantification of the p150 and p110 isoform. (D) Embryo weights from *Adar1^f/f^* and *Adar1^PKO^* embryos at E13.5 and E14.5. Dots represent individual mice (5 litters). (E) Volcano plot depicts DEGs detected by RNA-seq in E13.5 *Adar1^PKO^* versus *Adar1^f/f^* placentas. (F) GO analysis of significantly downregulated genes in *Adar1^PKO^* placentas at E13.5. (G) The interferome database V2.01I was used to characterize significantly upregulated ISGs specific to different Interferons (Type I, Type II, and Type III) in *Adar1^PKO^* placentas. (H) Heatmap represents relative expression of the top 50 upregulated genes in *Adar1^f/f^* and *Adar1^PKO^* placentas at E13.5. ISGs were indicated by the grey circle. (I) Relative mRNA expression of inflammatory genes in *Adar1^f/f^* (Gray) and *Adar1^PKO^* (Purple) placentas at E13.5 and E14.5 (n=6, 5 litters). In panels C, D, and I, Data are presented as mean ± SEM. **p* < 0.05; ns, non-significant*;* unpaired t test was used.


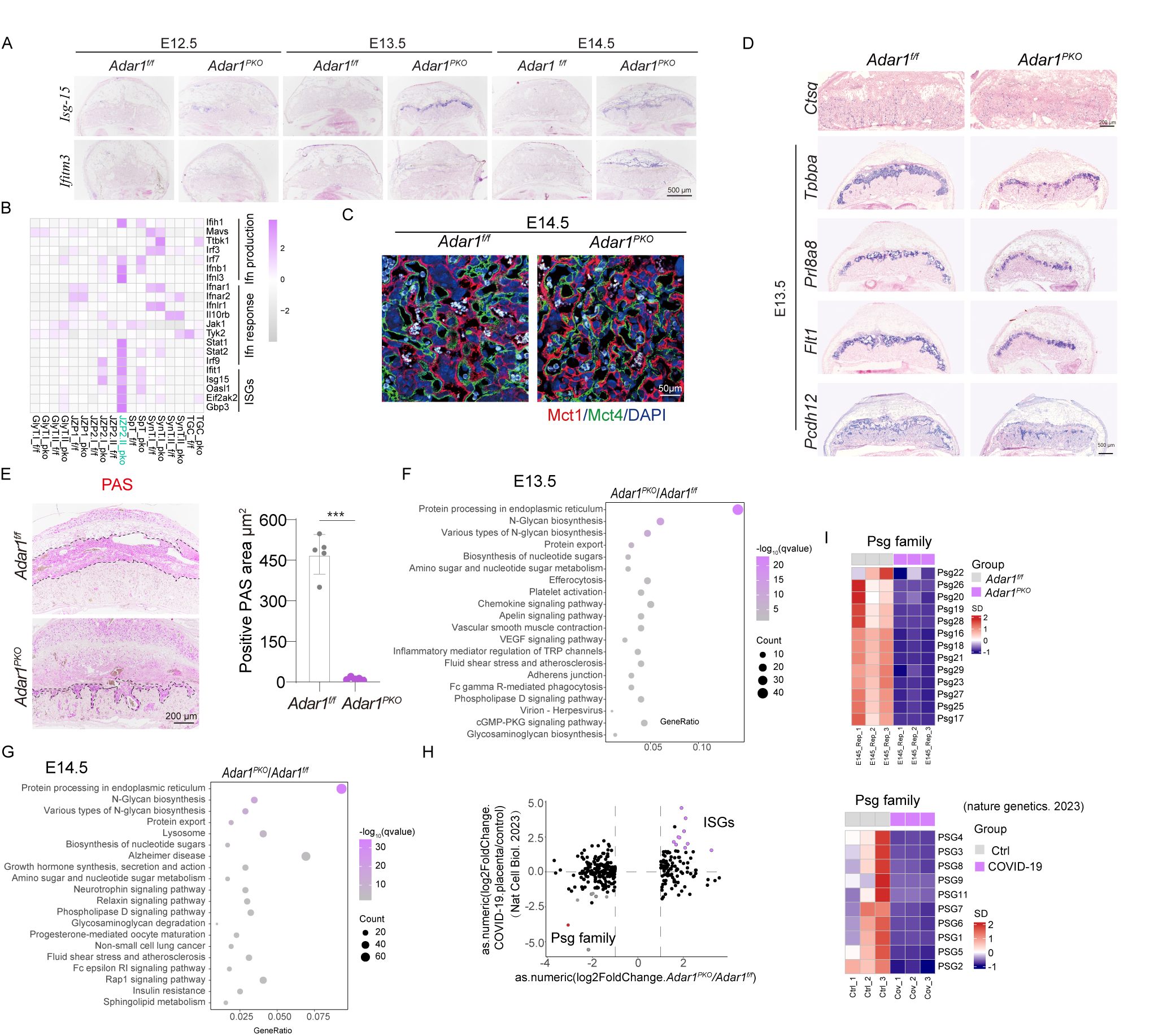


**Figure. S3. *Adar1* deletion in the placenta caused spontaneous IFN responses and placenta dysfunctions, related to Figure 2.** (A) ISH analysis of *Isg15* and *Ifitm3* in *Adar1^f/f^* and *Adar1^PKO^* placentas from E12.5 to E14.5. (B) Heatmap compares the relative expression of the upregulated genes associated with IFN signaling pathways in trophoblast populations from *Adar1^f/f^* and *Adar1^PKO^* placentas at E14.0. (C) Immunofluorescence staining for Mct1 and Mct4 in *Adar1^f/f^* and *Adar1^PKO^* placentas at E14.5. (D-E) ISH analysis of *Ctsq*, JZ markers (D) and PAS staining (E) in *Adar1^f/f^* and *Adar1^PKO^* placentas at E14.5. Right panel (E), the quantification of PAS-positive areas (n=5, 5 litters). (F-G) GO enrichment analysis of significantly downregulated genes in *Adar1^PKO^* placentas at E13.5 (F) and E14.5(G). (H-I) Scatter plot (H) and heatmap (I) showing the correlation of PSG family gene expression changes between mouse *Adar1^PKO^* placentas and human COVID-19-infected placentas (ID: E-MTAB-11749). In panel E, Data are presented as mean ± SEM. ****p* < 0.001; unpaired t test was used.


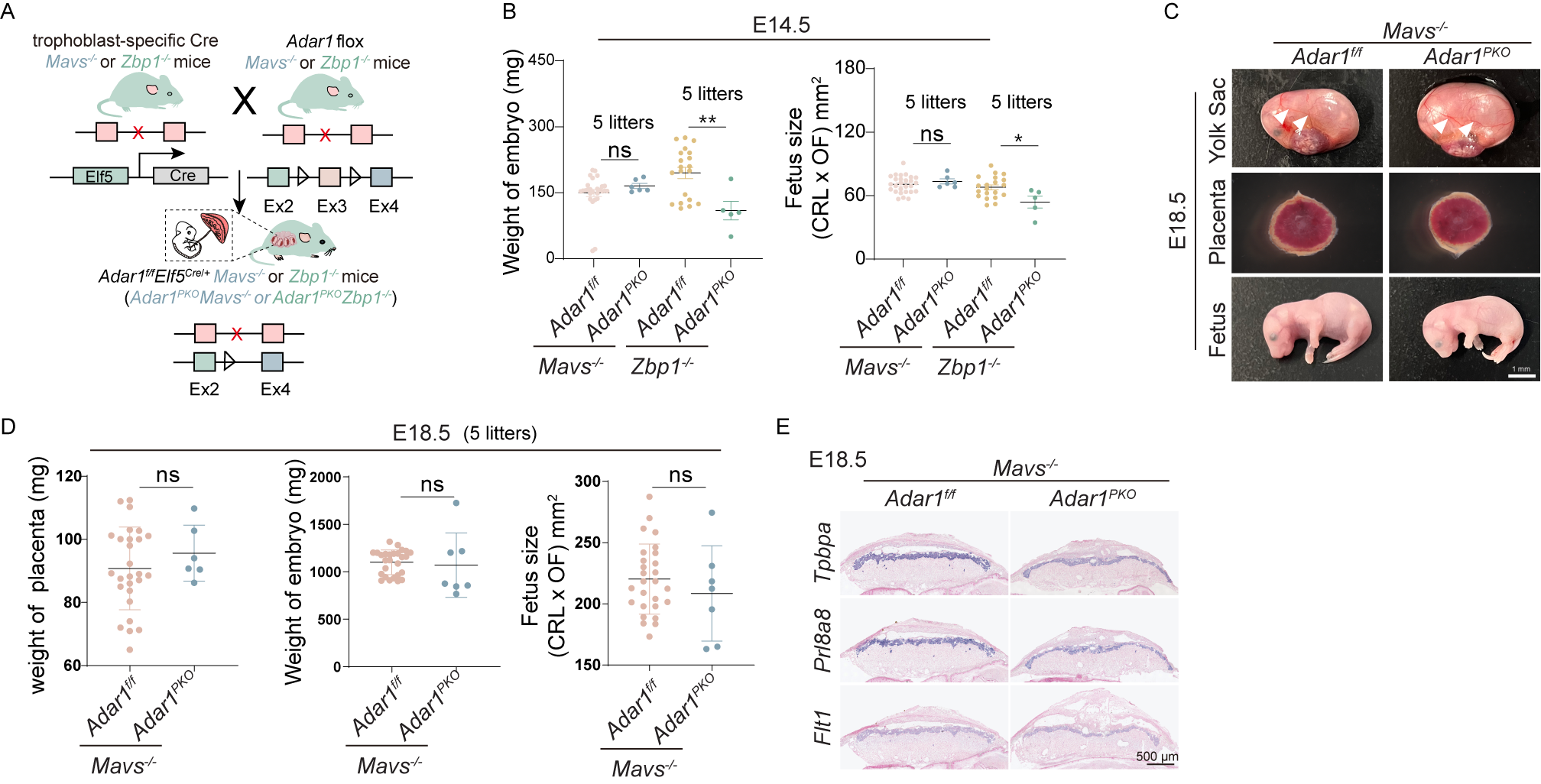


**Figure. S4. Deletion of *Mavs*, but not *Zbp1*, completely rescues embryonic mortality of *Adar1^PKO^* mice****, related to Figure 3.** (A) Schematic diagram illustrates the mating strategy to generate *Adar1^PKO^* with additional *Mavs* or *Zbp1* deletion. (B) Weight and size (measured by crown-rump length (CRL) and occipito-frontal (OF) diameter) of the indicated embryos at E14.5. Dots represent individual embryos (5 litters). (C) Stereomicroscopic images of the yolk sac, placenta, and fetus at E18.5. Arrowheads, yolk sac vasculature. (D) Scatter plot of placental weight, embryo weight, and fetus size at E18.5 (5 litters). (E) ISH analysis of JZ markers in *Adar1^f/f^Mavs^-/-^* and *Adar1^PKO^Mavs^-/-^* placentas at E18.5. In panels B and D, Data are presented as mean ± SEM. **p* < 0.05, ***p* < 0.01; ns, non-significant; unpaired t test was used.


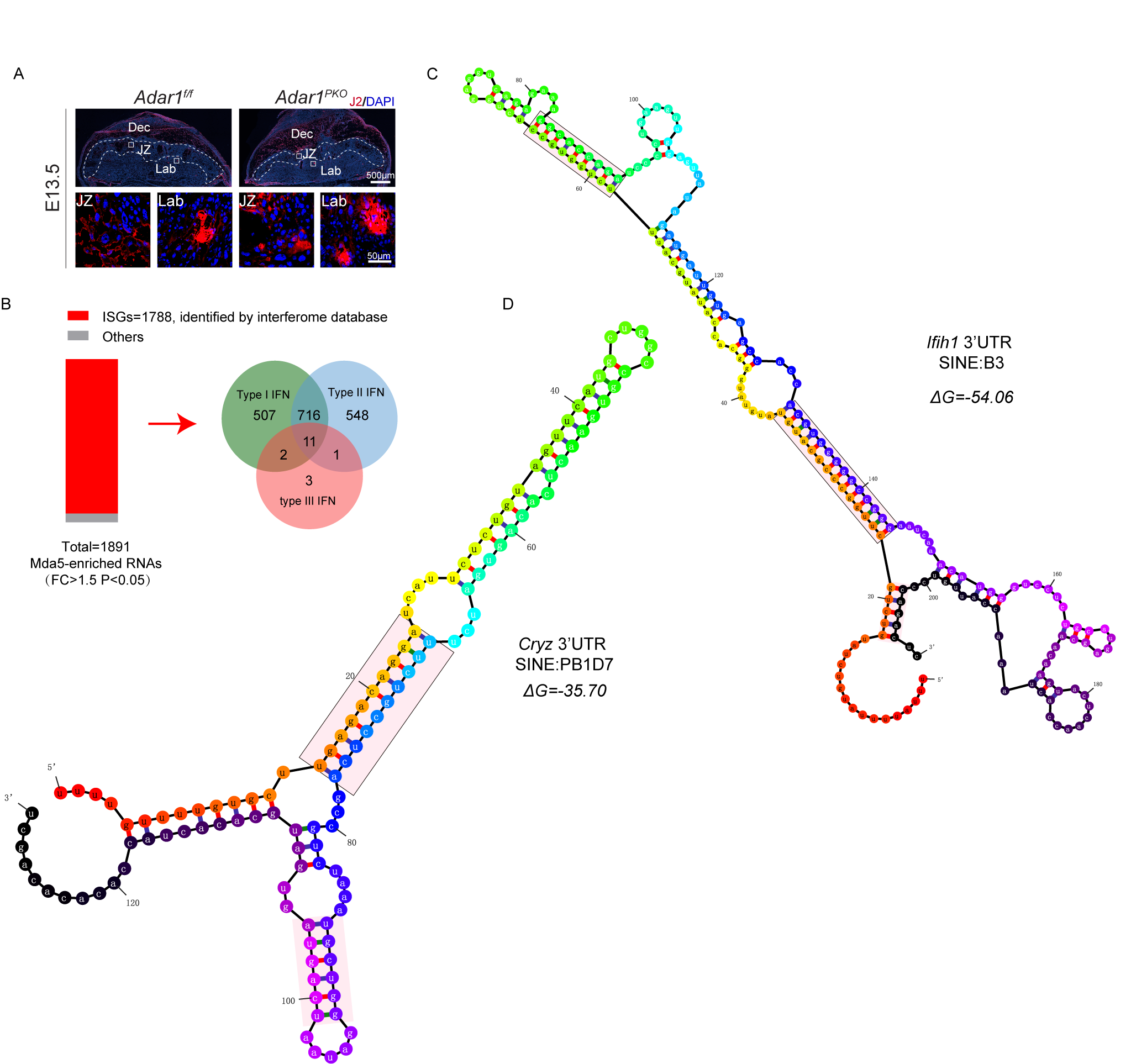


**Figure. S5. Analysis of dsRNA and interferon responses in *Adar1^PKO^* placentas and structural predictions of dsRNA elements, related to Figure 4.** (A) J2 immunofluorescence staining for dsRNA in E13.5 *Adar1^f/f^* and *Adar1^PKO^* placentas. (B) The interferome database was used to determine the number of ISGs specific to different types of IFNs within MDA5-RIP enriched RNAs in *Adar1^PKO^* placentas. (C-D) Predicted secondary structure (RNA-fold) of the SINE element within the 3'UTR of *Ifih1* (C) and *Cryz* (D) mRNA indicating the potential formation of a stable dsRNA structure. Pink boxes outline putative MDA5-binding sites.


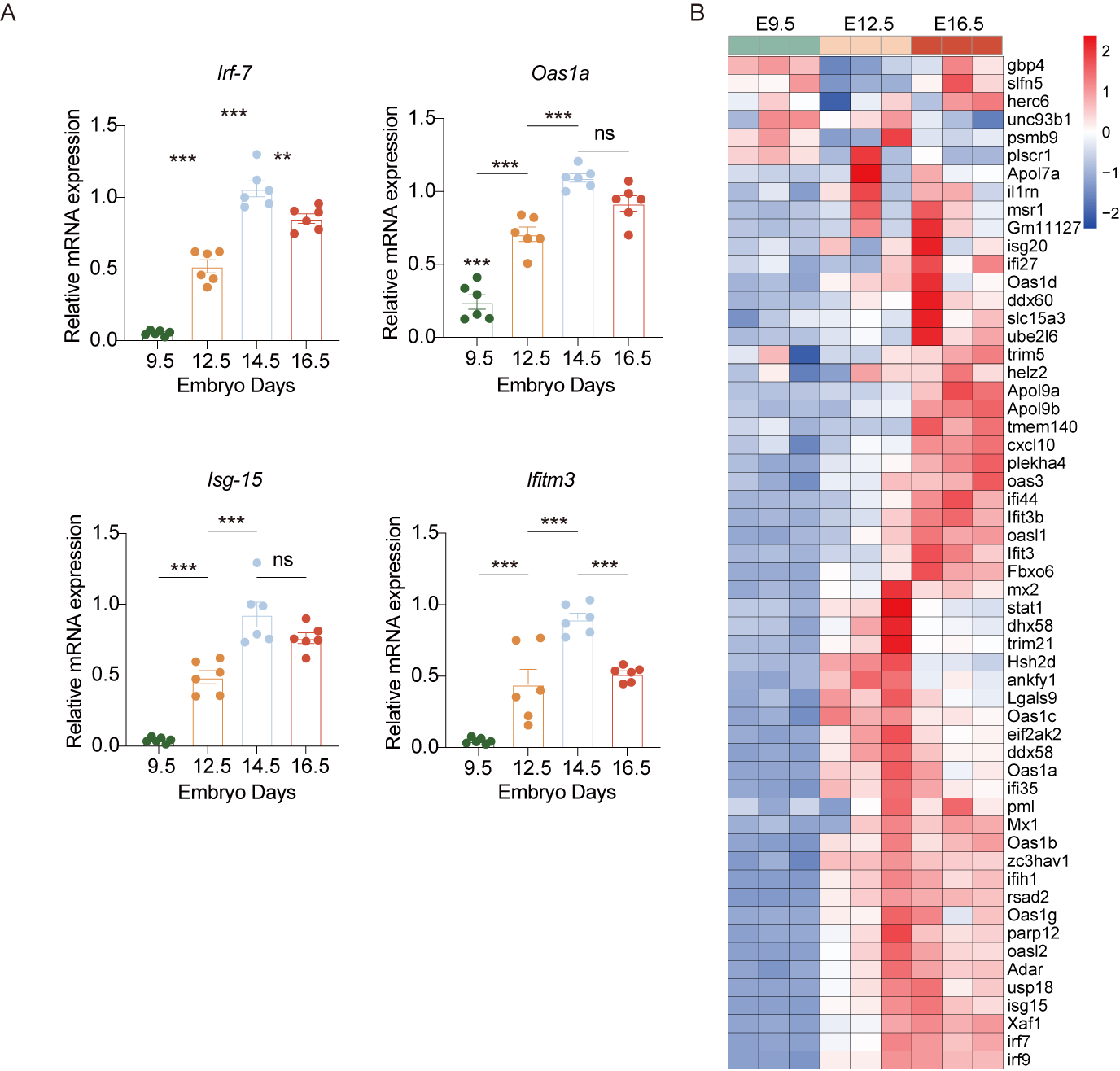


**Figure. S6. The placenta constitutively expresses IFNs and ISGs under physiological conditions,** **related to Figure 4.** (A) RT–qPCR analysis showing the relative expression of ISGs in wild-type placental tissues from E9.5 to E16.5 (n=6, 6 litters). Data are presented as mean ± SEM. **p* < 0.05, ***p* < 0.01, ****p* < 0.001; ns, non-significant; unpaired t test was used. (B) Heatmap represents expression levels of ISGs in the placenta from E9.5 to E16.5 detected by RNA-seq.


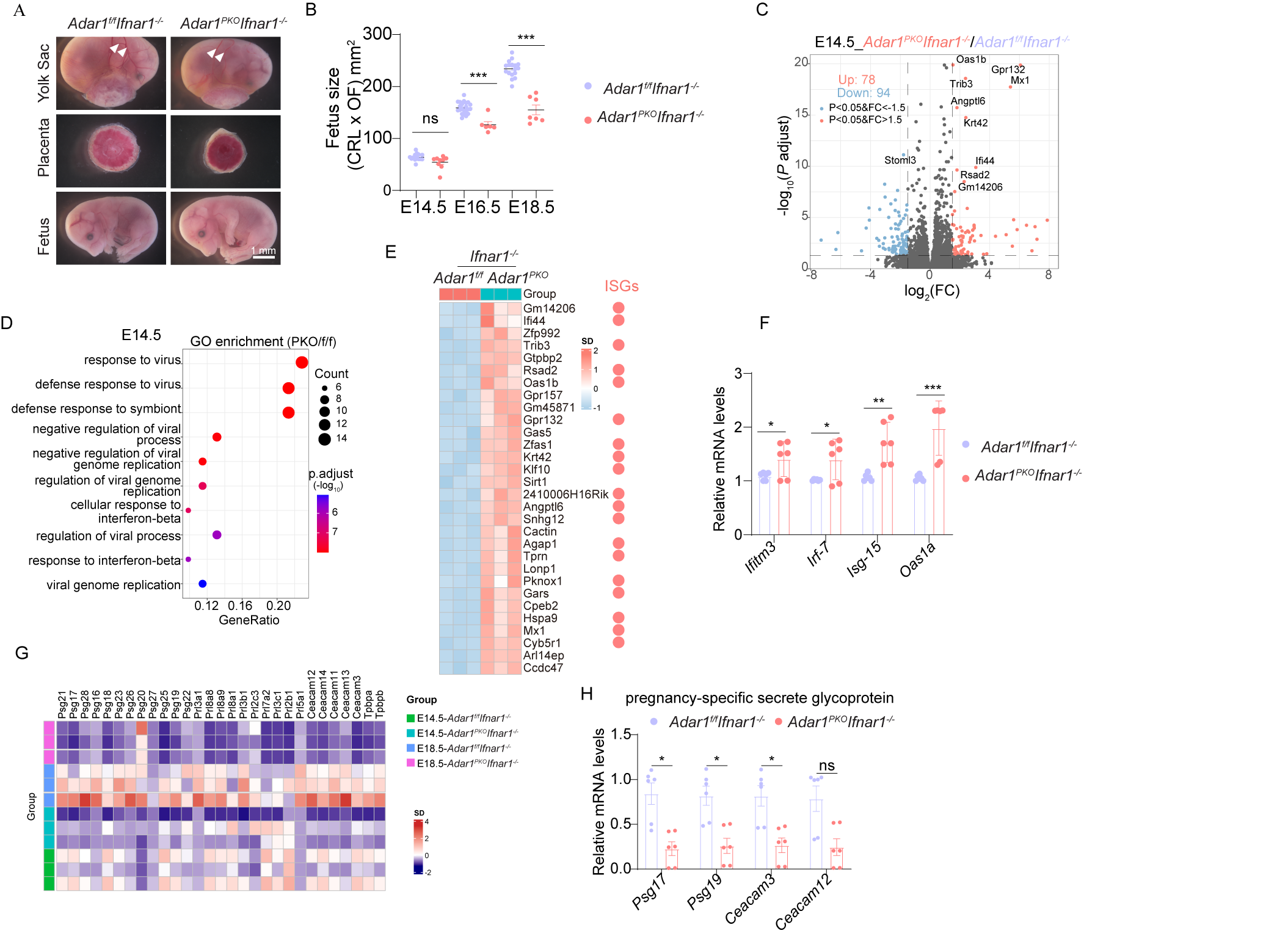


**Figure. S7. *Ifnar1* deletion fully rescues the embryonic lethality of *Adar1^PKO^* mice, related to Figure 5.** (A) Stereomicroscopic images of the yolk sac, placenta, and fetus from *Adar1^f/f^Ifnar1^-/-^* and *Adar1^PKO^Ifnar1^-/-^* embryos at E16.5. Arrowheads, yolk sac vasculature. (B) Fetal size is measured by CRL and OF diameter from *Adar1^f/f^Ifnar1^-/-^* and *Adar1^PKO^Ifnar1^-/-^* embryos at E14.5, E16.5, and E18.5, respectively (5 litters). (C) Volcano plots depict differential expression genes detected by RNA-seq of whole *Adar1^f/f^Ifnar1^-/-^* and *Adar1^PKO^Ifnar1^-/-^* placentas at E14.5. (D) GO analysis of differentially expressed genes in *Adar1^PKO^Ifnar1^-/^*^-^ placentas at E14.5. (E) Heatmap represents relative expression of the top 30 upregulated genes in *Adar1^f/f^Ifnar1^-/-^* and *Adar1^PKO^Ifnar1^-/-^* placentas at E14.5. Each gene belonging to ISGs is indicated by the orange circle as the Interferome dataset verified. (F) Relative mRNA expression of ISGs in *Adar1^f/f^Ifnar1^-/-^* and *Adar1^PKO^Ifnar1^-/-^* placentas at E14.5 (n=6, 5 litters). (G) Heatmap represents differentially expressed genes coding PSGs in *Adar1^f/f^Ifnar1^-/-^* and *Adar1^PKO^Ifnar1^-/-^* placentas at E14.5 and E18.5. (H) RT–qPCR analysis of relative PSG mRNA expression in *Adar1^f/f^Ifnar1^-/-^* and *Adar1^PKO^Ifnar1^-/-^* placentas at E14.5 (n=6, 5 litters). In panels B, F, and H, Data are presented as mean ± SEM. **p* < 0.05, ***p* < 0.01, ****p* < 0.001; ns, non-significant; unpaired t test was used.


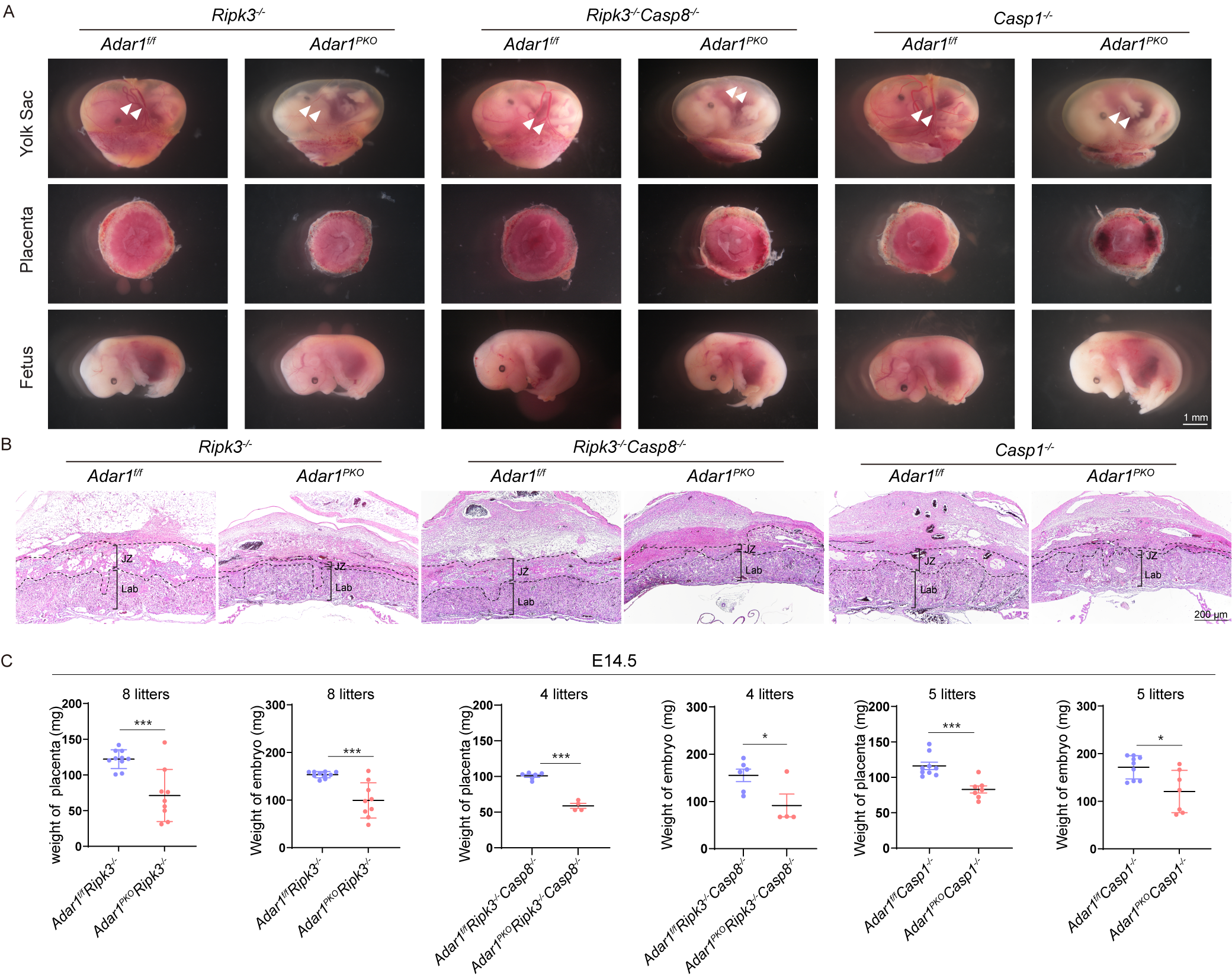


**Figure. S8. Blocking cell death did not mimic the effect of *PKR* deficiency in the *Adar1^PKO^* placenta.** (A) Stereomicroscopic images of the yolk sac, placenta, and fetus from the indicated genotypes at E14.5. Arrowheads, yolk sac vasculature. (B) H&E staining of placental sections from the indicated genotypes at E14.5. (C) Placental and Embryonic weights of the indicated genotypes at E14.5. Dots represent individual mice. In panel C, Data are presented as mean ± SEM. **p* < 0.05, ****p* < 0.001; unpaired t test was used.


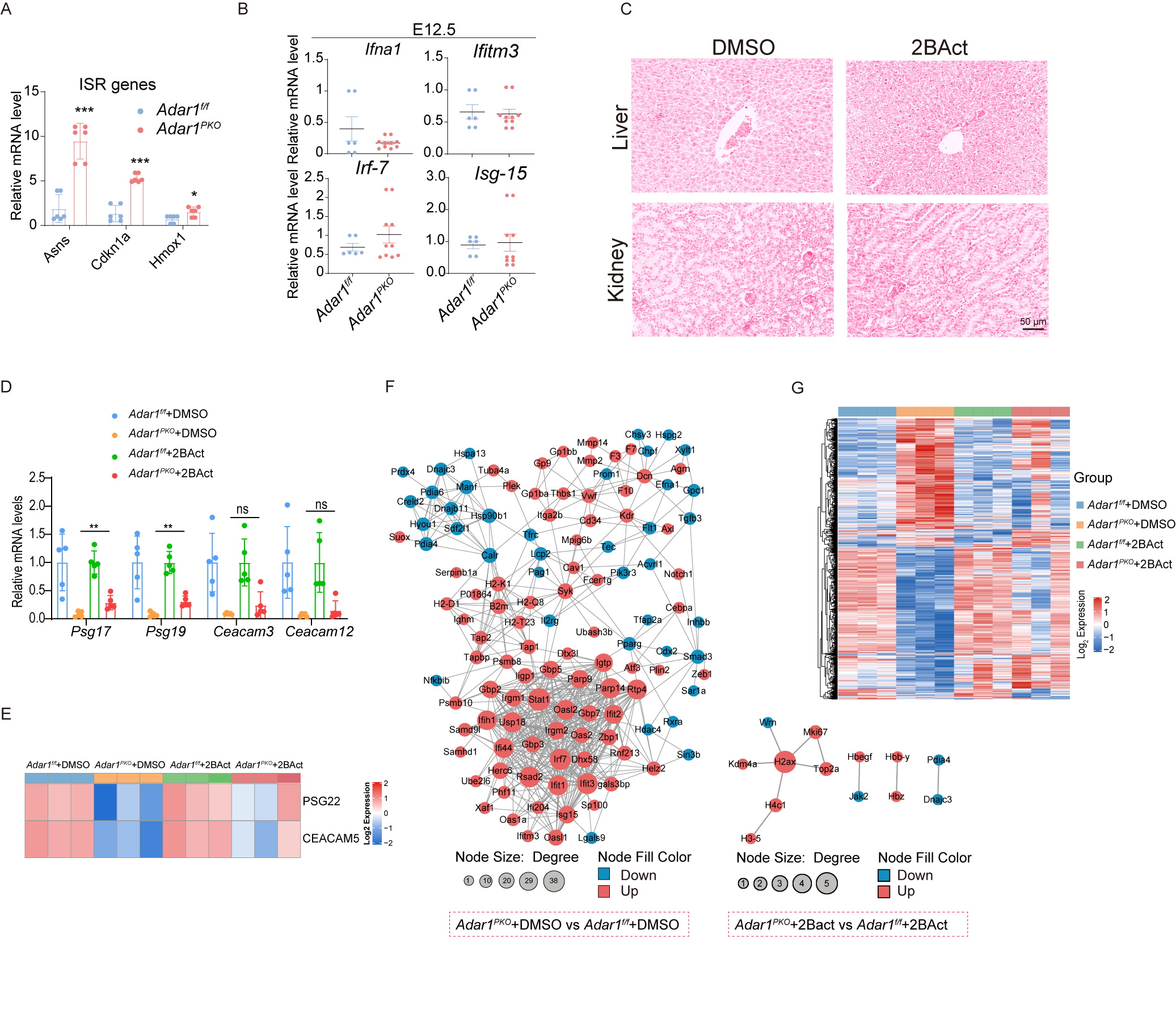


**Figure. S9. 2BAct treatment alleviated the pathology of *Adar1^PKO^* mice, related to Figure 7.** (A-B) RT–qPCR analysis of the mRNA expression of ISR genes in (A), IFN, and ISGs in (B) from *Adar1^f/f^* and *Adar1^PKO^* placentas at E14.5 or E12.5. (C) H&E staining of liver and kidney from *Adar1^PKO^* dams treated with DMSO or 2BAct. (D) RT–qPCR analysis of PSGs and Ceacams expression (n=5, 5 litters). (E) Heatmap of differentially expressed PSG22 and CEACAM5 in *Adar1^f/f^* and *Adar1^PKO^* placentas treated with 2BAct or DMSO. (F-G) PPI network (F) and heatmap (G) of differentially expressed proteins in *Adar1^f/f^* and *Adar1^PKO^* placentas treated with 2BAct or DMSO. In panels A, B, and D, Data are presented as mean ± SEM. **p* < 0.05, ****p* < 0.001; unpaired t test was used.

**
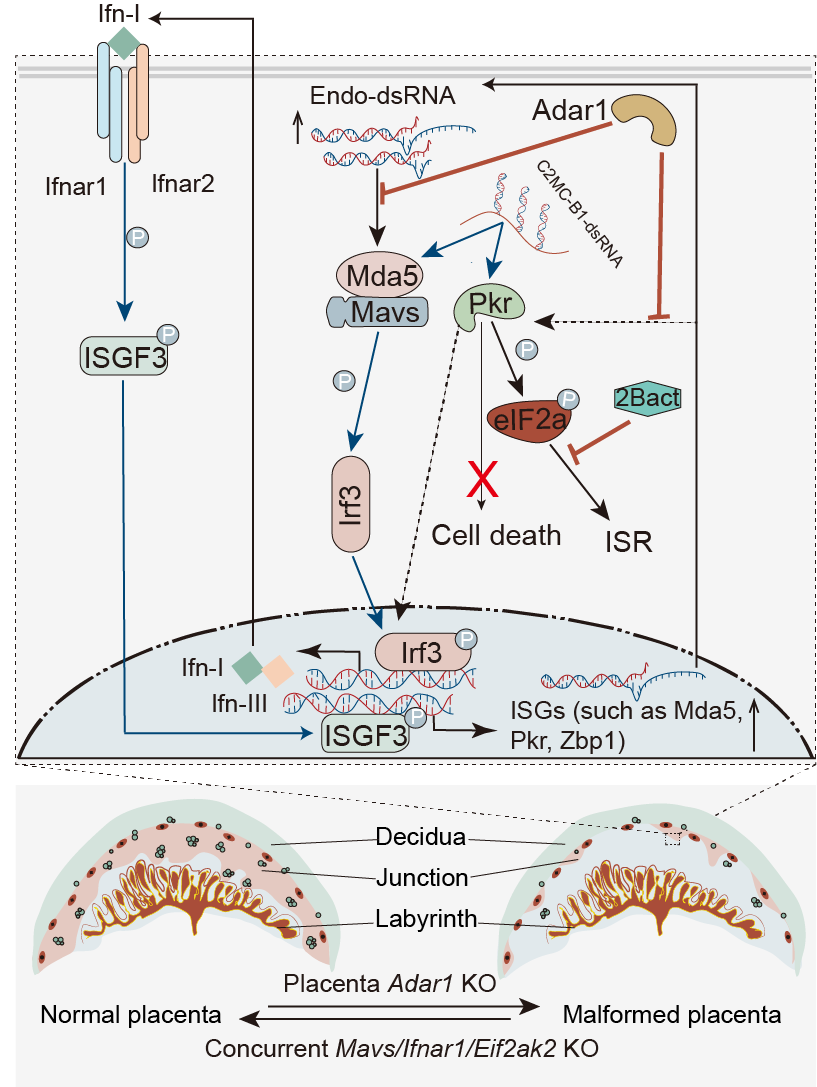
Figure. S10.** Schematic illustration showing ADAR1 as a placental innate immune rheostat sustaining the homeostatic balance of intrinsic interferon response at the maternal-fetal interface. ADAR1 fine-tunes the unique spatially resolved dsRNA-MAVS-IFN-PKR-ISR signaling in the placenta by restricting ISG-3′UTR-dsRNA accumulation.

**Table S1. Genotyping primers (F: forward primer; R: reverse primer)**

| Primers | Sequence (5’-3’) | |
| --- | --- | --- |
| *Elf5-cre* | F | GGGTTGACCGGAGGTTAGTG |
| *Elf5-cre* | R | GTTCGAACGCTAGAGCCTGT |
| *Elf5-cre* wt | F | GGGTTGACCGGAGGTTAGTG |
| *Elf5-cre* wt | R | GTTGCCACAAGACCATCCCT |
| *Adar1 flox* | F | ACAGTCTTCTGGGATAGCACTCCTG |
| *Adar1 flox* | R | ATATCAGAACCAGCTTCTCTGGGC |
| *Adar1* ko | F | ACAGTCTTCTGGGATAGCACTCCTG |
| *Adar1* ko | R | ATGGCCGACTATGATACATCTGC |
| *Caspase8* | F | TGTCTGGAATATGGTAGATGCAGT |
| *Caspase8* | R | AGTCTCACAAGTCACTTCACACA |
| *Caspase1* | F | GAGTGACAGAGATGGTGGC |
| *Caspase1* | R | TGAGACACACAGCAGAGCC |
| *Mavs* | F | AGCCAAGATTCTAGAAGCTGAGAA |
| *Mavs* Neo | R | GTGGAATGTGTGCGAGGCCAGAGGC |
| *Mavs* | R | TAGCTGTGAGGCAGGACAGGTAAGC |
| *Eif2ak2* ko | F | GGCTACCAGCCAAGCTCTAAGTTC |
| *Eif2ak2* ko | R | GACATGCTGGCAACCTAACTCTG |
| *Eif2ak2* wt | F | GTGATTCGTGTGTGAGACGAGAAG |
| *Eif2ak2* wt | R | GACATGCTGGCAACCTAACTCTG |
| *Ripk3* | F | GGCGTGTAGGAAGAAGATAT |
| *Ripk3* | R | ATCCAGCAGAATGTTAGAGG |
| *Zbp1*ko | F | AGGACCCTCTTCCCACTAAG |
| *Zbp1*ko | R | TCTTGGCCTTCCTGAGTGG |
| *Zbp1* wt | F | TGGAAGATCTACCACTCACGTC |
| *Zbp1* wt | R | GGCAATGCGTTCATACACA |
| *Ifnar1* | F | CCAGGAGATACATTCTTAACC |
| *Ifnar1* | R | GAACAATATAGCCAGCCATG |

**Table S2. qPCR primer sequences**

| Target genes | Sequences (5’-3’) | |
| --- | --- | --- |
| *Ifnb1* | F | CAGCTCCAAGAAAGGACGAAC |
|  | R | GGCAGTGTAACTCTTCTGCAT |
| *Adar1* | F | GCCAAAGACAGTGGTCAACCAG |
|  | R | GAACAAGGATGTTGCTGAGGAGC |
| *Ifna1* | F | CTGCTGGCTGTGAGGAAATA |
|  | R | CACATTGGCAGAGGAAGACA |
| *Ifnl2* | F | AGCTGCAGGTCCAAGAGCG |
|  | R | GGTGGCCAGGGCTGAGTCA |
| *Irf-7* | F | TCTCGGCTTGTGCTTGTCTA |
|  | R | TTGGGAGTTGGGATTCTGAG |
| *Isg-15* | F | TAATTCCAGGGGACCTAGAGC |
|  | R | ACACCAGGAAATCGTTACCC |
| *Oas1a* | F | GGAGGCGGTTGGCTGAAGAGG |
|  | R | GAACCACCGTCGGCACATCC |
| *Ifitm3* | F | CTGCTGCCTGGGCTTCATAG |
|  | R | GGATGCTGAGGACCAAGGTG |
| *Tnf-α* | F | GGAACACGTCGTGGGATAATG |
|  | R | GGCAGACTTTGGATGCTTCTT |
| *Il-6* | F | CTGCAAGAGACTTCCATCCAG |
|  | R | AGTGGTATAGACAGGTCTGTTGG |
| *Il-1* | F | GAAATGCCACCTTTTGACAGTG |
|  | R | TGGATGCTCTCATCAGGACAG |
| *Aldh1* | F | GGGTCACACTGGAGCTAGGA |
|  | R | CTGGCCTCTTCTTGGCGAA |
| *Prl8a8* | F | ACCCACGGATGGAAACATTTG |
|  | R | TGCAGCTCTGAAAACAATCTCAT |
| *Psg17* | F | TCGTGCTGTCACCCTCTTGA |
|  | R | TACTCCGTGCATAGCCCATGA |
| *Psg19* | F | TGCTGTGACGCTTTCAACTCT |
|  | R | GGCCACTGATGATAGACTCTGTC |
| *Ceacam12* | F | GGTACAAAGGAGGTAAACTGCTC |
|  | R | CGCATTATTTCACGTCTTGAGGT |
| *Ceacam3* | F | TGCCCACCACTACCCAAGT |
|  | R | CCCCTTTGTACCAGATAAAGCC |
| *Ifih1* | F | GGAATGCCCATGAGGTATTG |
|  | R | AGCTTGCCACATTGCATTG |
| *Cryz* | F | GAGGGCTATTCGAGTGTTTGAG |
|  | R | GGCGTGGACTTTGATTAGGAC |
| *Gapdh* | F | AGGTCGGTGTGAACGGATTTG |
|  | R | TGTAGACCATGTAGTTGAGGTCA |
| *Ctsq* | F | GTGTTTCAGCATTTGATCCCAGT |
|  | R | GTCAGCAAACCCATTTAATCCCA |
| *Plf/Prl2c2* | F | AACGCAGTCCGGAACGGGG |
|  | R | TGTCTAGGCAGCTGATCATGCCA |
| *Slc16a1* | F | TCGCAGCTTCTTTCTGTAACAC |
|  | R | TCATAGTCAGAGCTGGGTTCAA |
| *Slc16a3* | F | CCACAAGTTCTCCAGTGCCATTG |
|  | R | CGCCAGGATGAACACGTACATG |
| *Syn-a* | F | ATGGTTCGTCCTTGGGTTTTC |
|  | R | GTGTTGAGTGAGGTTTACCAGG |
| *Syn-b* | F | CAGCTGACACCCTCATTAAACA |
|  | R | ATCCAGAAATGGGAATGAAG TG |
| *Tpbpa* | F | ACTGGAGTGCCCAGCACAGC |
|  | R | GCAGTTCAGCATCCAACTGCG |
| *Pcdh12* | F | GAAGAGCTGTCGAGCCTGTT |
|  | R | GTGAGGGGCAATGACAATCT |
| *Asns* | F | GAAACTCTTCCCAGGCTTTGAC |
|  | R | TTCAGCAGAGAGGCAGCAAC |
| *Cdkn1a* | F | CCTGGTGATGTCCGACCTG |
|  | R | CCATGAGCGCATCGCAATC |
| *Hmox1* | F | AAGCCGAGAATGCTGAGTTCA |
|  | R | GCCGTGTAGATATGGTACAAGGA |
| *Mad2l1* | F | AATTTTCCGGTGAAGAAAGC |
|  | R | AGCTTTGATCCCTTCTGCTG |
| *Rpa1* | F | CTCAGAGGGCTGTGTGTGAA |
|  | R | AGACAAAAAGGTGCCACCAC |
| *Rbbp4* | F | TTCTCCTGGAATCCCAATGAGCC |
|  | R | AAAACATACATCTTTACAACTTGGTGG |
| *Pvt1* | F_a_ | ACTTAGCATTCCCAGAGCCC |
|  | F_b_ | CCATGACTGGGAAAAACCTCG |
|  | R | TGGAGGGCATCTTCTTACCG |
|  |  |  |
|  |  |  |

**Table S3. Primers for ISH probe preparation**

| Target genes | Sequences (5’-3’) | |
| --- | --- | --- |
| *Ifitm3* | F | GACAGCCCCCAAACTACGAA |
|  | R | CTTAGCAGTGGAGGCGTAGG |
| *Isg15* | F | CGATTTCCTGGTGTCCGTGA |
|  | R | AGCCAGAACTGGTCTTCGTG |
| *Ctsq* | F | GTCCTCCCAGAAAGTGAAGATG |
|  | R | TCACACAGTAGGGTATTGG |
| *Pcdh12* | F | ACAGTTTCCCAAAGACGAGCA |
|  | R | GACCGTTGTTTCCTGAGTCCA |
| *Sflt1* | F | GAGAGCATCTCTCAGCGCAT |
|  | R | GGCCCAAGAAGTGACTACCC |
| *Prl8a8* | F | ATAACTCCGATGAAGC |
|  | R | TCAATAGTTATGTATTGAA |
| *Tpbpa* | F | CAACATAGAAATGAGTGCC |
|  | R | ACACAGTTAATTATGCCTT |

| Target genes | Sequences (5’-3’) |
| --- | --- |
| *Isg15*_m_pr1_SpIR | TGCGTCAGAAAGACCTCATATCCTCTATGATTACTGACTGCGTCTATTTAGTGGAGCCGCCCCTATCTTCTTTTCTTAAGCGTGTCTACAGTC |
| *Isg15*_m_pr2_SpIR | AGAAAGACCTCATAGATGTTTCCTCTATGATTACTGACTGCGTCTATTTAGTGGAGCCGCCCCTATCTTCTTTTGTCTACAGTCTGCGTC |
| *Zbp1*_m_pr1_SpIR | TCATCAAGGCTAGGCTGTCCTCTATGATTACTGACTGCGTCTATTTAGTGGAGCCGCCCCTATCTTCTTTTTCCAGGAATCTTAATATTCTT |
| *Zbp1*_m_pr2_SpIR | TTATTTCTCATGGAATACAGGTCCTCTATGATTACTGACTGCGTCTATTTAGTGGAGCCGCCCCTATCTTCTTTTCATAGCTCAGAAGGTGC |
| *Zbp1*_m_pr3_SpIR | CGGTAAAGGACTTGATTGAGTCCTCTATGATTACTGACTGCGTCTATTTAGTGGAGCCGCCCCTATCTTCTTTCTGTCCTCCTTCTTCAGG |
| *Adar1*_m_pr1_SpIR | GAAATCACAGGTCTGACTAGTCCTCTATGATTACTGACTGCGTCTATTTAGTGGAGCCGCCCCTATCTTCTTTCACTCTGCTCTATCAGGTT |
| *Adar1*_m_pr2_SpIR | ATCTTCTCCTTGATTTCAGCTCCTCTATGATTACTGACTGCGTCTATTTAGTGGAGCCGCCCCTATCTTCTTTCATTGAACAGATAGTCACAG |
| *Adar1*_m_pr3_SpIR | TATCTGGGATGTCATCTGTGTCCTCTATGATTACTGACTGCGTCTATTTAGTGGAGCCGCCCCTATCTTCTTTCTGTGTGGATACTATTCAAGT |

**Table S4. Primers for SCRINSHOT probe preparation**
